# The Development of a Strategic Prioritisation Method for Green Supply Chain Initiatives

**DOI:** 10.1371/journal.pone.0143115

**Published:** 2015-11-30

**Authors:** S. Maryam Masoumik, Salwa Hanim Abdul-Rashid, Ezutah Udoncy Olugu

**Affiliations:** Centre for Product Design and Manufacturing (CPDM), Department of Mechanical Engineering, Faculty of Engineering, University of Malaya, Kuala Lumpur, Malaysia; Southwest University, CHINA

## Abstract

To maintain a competitive position, companies are increasingly required to integrate their proactive environmental strategies into their business strategies. The shift from reactive and compliance-based to proactive and strategic environmental management has driven companies to consider the strategic factors while identifying the areas in which they should focus their green initiatives. In previous studies little attention was given to providing the managers with a basis from which they could strategically prioritise these green initiatives across their companies’ supply chains. Considering this lacuna in the literature, we present a decision-making method for prioritising green supply chain initiatives aligned with the preferred green strategies alternatives for the manufacturing companies. To develop this method, the study considered a position between determinism and the voluntarism orientation of environmental management involving both external pressures and internal competitive drivers and key resources as decision factors. This decision-making method was developed using the analytic network process (ANP) technique. The elements of the decision model were derived from the literature. The causal relationships among the multiple decision variables were validated based on the results of structural equation modelling (SEM) using a dataset collected from a survey of the ISO 14001-certified manufacturers in Malaysia. A portion of the relative weights required for computation in ANP was also calculated using the SEM results. A case study is presented to demonstrate the applicability of the method.

## Introduction

In today’s competitive market, the companies are increasingly being encouraged to incorporate environmental strategies into their business strategies. The increased competition and globalisation have caused organisations to shift from local optimisation at the firm level towards the entire supply chain [1]. Hence the scope of environmental activities is extended beyond the firm’s internal borders [2]. This operational transition in environmental practice provides companies with opportunities for the broader development of sustainability [3]. In this respect, integrating environmental strategies into supply chain management has become a subject of growing interest among academics and practitioners.

Environmentally conscious thinking in supply chain management offers a long list of green initiatives in various operational areas across the value chain from the raw material supply to product usage and even further to post-use processes including reverse logistics, product recovery and recycling [1, 2, 4, 5]. However, the resource constraint does not allow companies to implement all these practices in an effective way [6]. That is why managers have to strategically prioritise their green supply chain initiatives (GSCIs) to gain as many environmental and competitive benefits as possible.

The pressures from external stakeholders, such as regulators, customers, suppliers, competitors, community groups and the media, force organisations to implement several determinant and regulatory-driven green practices. However, there are still numerous voluntary environmental activities that can be initiated by companies depending upon their competitive position and their internal resources [7]. This provides the opportunity for making strategic choices concerning those GSCIs that can be initiated proactively.

To implement strategic planning concerning green supply chain initiatives, business managers are required to simultaneously consider external pressures, internal competitive drivers and firm’s key resources.

However, there has been little discussion in the literature that shows how external pressures and internal resources interactively can affect managers’ decisions to prioritise green strategies and initiatives [8]. Most of the previous research attempting to develop a decision-making framework for prioritising GSCIs is more focused on the operational level than the strategic level (See e.g., [9–11]).

Considering this lacuna in the literature, the present study aims to develop a decision-making method for prioritising green strategies and GSCIs while considering both external and internal strategic factors. This decision method will help business managers answer the following questions while they are developing their strategic environmental plan:

1. What will be the priorities of green strategies if the company aims to satisfy the following objectives simultaneously?
  - Meeting the demands of external stakeholders
  - Achieving the firm’s desired competitive advantages
  - Optimising the exploitation of the firm’s key resources
2. What will be the priorities of GSCIs if the company aims to adopt the prioritised green strategies?

To develop a more reliable method, the present study is devoted to establishing the link between empirical studies and the approach of quantitative decision-making modelling that has only been carried out in a small number of previous studies [12]. For this purpose we used the concept of analytic network process (ANP) modelling [13] for structuring our decision framework and then conducted an empirical study to explore the relationships between the factors and the relative weights of variables required for solving the problem using the ANP technique. We have analysed the causal relationship model by applying the method of partial least square-based structural equation modelling (PLS-SEM) [14] using the dataset generated from our survey of Malaysian ISO 14001-certified manufacturers.

To demonstrate the applicability of the proposed method, we tested the method using the case study of a manufacturer from the electronics industry in Malaysia.

## Theoretical Background

Recently, researchers and practitioners have shown an increased interest in the topic of green supply chain drivers, pressures and practices. There have been several empirical studies in the literature analysing the determinant factors and their impacts on the development of green initiatives in supply chains [15–21].

Most previous studies assumed that there is a direct relationship between external drivers and green supply chain initiatives. Most researchers referred to external drivers as institutional pressures imposed by regulatory bodies [15, 16, 18, 22–25], customers [15, 16, 18, 22–26], competitors [24, 25, 27], and society [18, 25, 26]. However, when it comes to internal drivers, there is no such consensus regarding the operationalization of the concept. “Supplier’s readiness in terms of having awareness, and know-how” [23], “external knowledge exchange” [28], “purchasing’s environmental capabilities” [20], and “waste reduction resources” [29], are some examples of internal drivers in previous studies, which can be categorized under the category firm’s key resources. Firm’s competitive advantage is another category of internal drivers that has been addressed in published articles in terms of cost reduction [15, 18, 22], competitive reasons [30, 31] and organization’s value [18].

From the literature review, it can be seen that the external drivers have received more attention from researchers compared to the internal drivers. Examining the impact of firms’ key resources as an internal driver or enabler to drive the green initiatives is placed in second rank. Although there are several arguments proving that the implementation of green initiatives generates competitive value for companies [32–34], few studies considered the firm’s desire to gain competitive advantages as an internal factor to drive GSCM.

Additionally, several attempts have been made to evaluate environmentally related practices by applying the ANP/AHP techniques. So far, however, these studies paid more attention to the operational level of decision making than the strategic level.

Previous studies suggested various independent decision factors that impact the priority vector of green supply chain initiatives. These include internal factors, such as “time period” [9, 35], “product life cycle stages” [10], “performance criteria” [10, 11, 36] and “firm’s readiness to implement GSCM” [35]; and external factors, such as “regulatory environment” [9], “customer requirements” [37, 38] and “non-government organization’s influence” [38].

In reviewing the internal decision factors, it can be seen that the internal strategic factors associated with competitive advantage have been partially addressed in a few studies. For example, Sarkis [10], and Buyukozkan and Cifci [11] considered “cost” as an independent factor that impacts on the priority vector of alternative green supply chain systems. Bai and Sarkis [39] also suggested economic performance as an influencing factor for ranking green supplier development programmes. Other elements associated with competitive advantage, such as “reputation and legitimacy” and “future positioning”, have been neglected in previous studies.

A firm’s key resources constitute another internal strategic factor that has been rarely addressed in the literature. In its general form, the influence of a firm’s key resources on the prioritisation decision for green supply chain initiatives has been presented as the readiness of a firm’s operational process to contribute to green programmes [35].

With regards to external strategic factors, a few studies include institutional pressures, such as regulatory pressures [9], customer pressures [37, 38], and society pressures [38] in their decision model. However, competitive pressure has been ignored in previous studies.

While the role of a firm’s environmental strategic approach to drive the implementation of green supply chain initiatives is emphasized in the literature [40, 41], most of the established decision models for ranking the green initiatives did not take this factor into account. In other words, they suggested the decision model for ranking green supply chain initiatives in various operational areas without considering the fact that the firm’s green strategic approach affects the scope of green supply chain improvement efforts.

Only a few studies paid attention to the role of green strategies in prioritising green supply chain initiatives. In this respect, the study conducted by Chen et al. [42] made an attempt to establish a decision model for green strategy selection. They suggested four types of green strategy, namely, risk-based, efficiency-based, innovation-based and closed loop strategy. However, they formulated the decision model for prioritising green strategies by taking a backward approach, which implies that the prioritisation of green strategies has been made based on the current environmental performance of the organisation. Researchers using this approach first identify the organisation’s green management perspective based on the environmental activities performed by the company and then obtain the priorities of green strategies and initiatives with respect to this perspective. The green initiatives introduced in their model include green design, green manufacturing, green purchasing and green marketing.

Because organisations have to adopt a proactive approach to gain competitive advantages, the model suggested in this paper will take a forward approach. In this approach, the company first decides which green strategy should receive the most focus while considering all the external and internal factors. Then, in the second stage the priorities of GSCIs to achieve these prioritised strategic goals will be determined. These priorities will be made based on two factors, the importance of the green initiatives to the fulfilment of the requirements of the green strategy and the current performance of the firm in each green initiative.

The first step in making the strategic prioritisation of GSCIs is to determine the green strategies preferences of the company. To do so, we applied two well-known theories, namely, the natural resource-based view (NRBV) [43–45] and institutional theory [46].

According to institutional theory, external pressures, by forcing companies to implement regulatory-driven practices, reduce the variation in firms’ environmental strategies. Meanwhile, the natural resource-based view (NRBV) suggests that the firm’s key resources providing differentiation can increase the variation of strategies [47].

Institutional theory emphasises the role of external pressures imposed on the organisation to develop green initiatives. The main sources of external pressures are regulatory bodies, customers, competitors, and society (in terms of NGOs, the media, and community groups) [25, 46]. The strategic factors associated with institutional pressures that are external to companies force them to implement some determinant environmental-related activities that are usually homogenous in the same industry.

According to the NRBV framework, there are three main green strategies, namely, pollution prevention, product stewardship, and clean technology, which can bring to companies the competitive advantages of cost reduction, reputation and legitimacy, and future positioning, respectively. The strategic factors of competitive advantages expected by companies can motivate them to incorporate several voluntary green initiatives in their environmental improvement programs. These voluntary green practices can provide companies the expected competitive advantages.

Each of the above-mentioned green strategies dictates a series of green initiatives in various operations of the supply chain based on the following definitions:

- Pollution prevention refers to reducing waste and emissions from the company’s current operations through the incremental improvement of the company’s existing products and processes.
- Product Stewardship relates to reducing the environmental impact of a company’s existing products and processes at every stage of a product’s life cycle (from supplying raw material, though the production processes, to product consumption and disposal of end-of-life products).
- Clean technology extends beyond a company’s existing products and business models. Companies adopting this approach use innovative technologies and make a disruptive change in their product and process design so they can gain benefits from future market opportunities.

By referring to the definition of these green strategies, adoption of these strategies can be realised through the various green initiatives across the supply chain classified in the following categories [27, 48]:

- Product design for the environment involves initiatives relating to the design of products for reuse, recycling, or recovery, the design of products for reducing emissions and other environmental design objectives.
- Greening upstream relates to using environmentally friendly materials, collaboration with suppliers in environmental objectives, and other upstream activities.
- Green production includes activities such as the optimisation of manufacturing processes to reduce waste, the consumption of materials and energy and the recycling of materials internally in the company.
- Greening downstream addresses environmentally friendly transportation, green packaging, and cooperation with customers in environmental objectives.
- Greening post-use refers to initiatives treating used products such as recovery activities for used or defective products/components or recycling from end-of-life products.

According to NRBV, key resources such as continuous improvement, stakeholder integration, and disruptive change enable companies to implement these green strategies. The strategic factors of a firm’s key resources can help managers make more realistic decisions on launching green initiatives with achievable performance targets.

As a consequence of applying these two theories, we introduced five clusters in our decision-making model. There are three clusters for the strategic factors that influence the prioritisation of green strategies, namely, institutional pressures, firm’s desired competitive advantages, and firm’s key resources. The other two clusters are green strategies and green supply chain initiatives.

## Research Methodology

**Fig 1:**
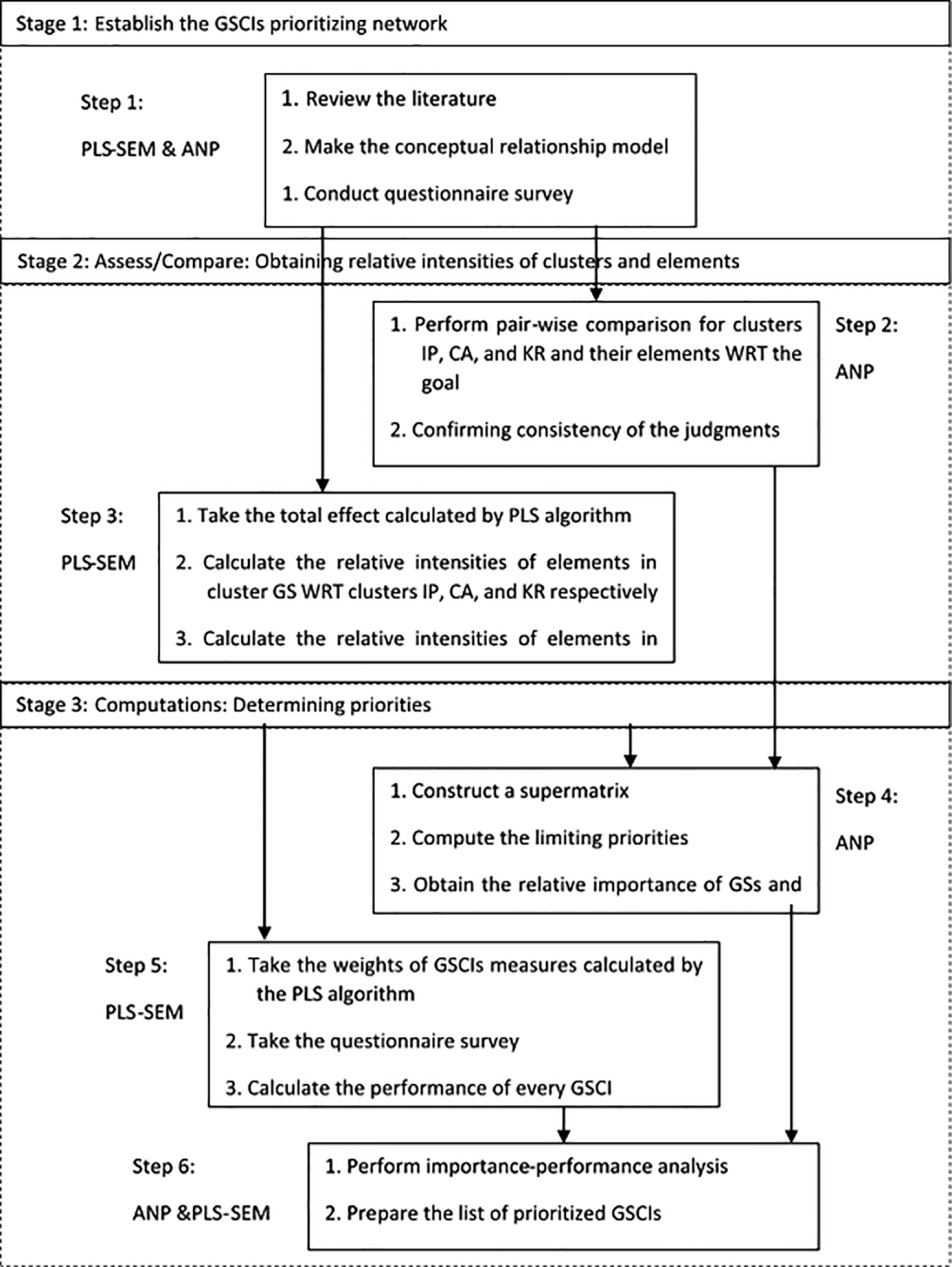
The proposed research process.

To develop a decision-making method for prioritising green strategies and initiatives, this study went through six steps within three main stages in the research process (see Fig 1).

### Establish the GSCIs prioritising network

The study begins with a review of the literature on green strategies and GSCIs and the factors that influence the decisions of firms’ managers in the prioritisation of green strategies and initiatives. The outcome of this step is a conceptual model of the causal relationship among the determinant factors, green strategies, green initiatives and performance.

To validate the developed conceptual model, we conducted a questionnaire survey among the ISO 14001-certified manufacturers in Malaysia. The manufacturing sector of Malaysia accounted for 24.9% of the gross domestic product (GDP) in 2012, and is considered to be the second largest contributor in Malaysia’s GDP [49]. Through the contribution of 66.5% in the value of total exports in the first quarter of 2014, it is also placed in first position in terms of the contribution to Malaysia’s export market. These facts show the importance of the manufacturing sector in respect of Malaysia’s economic growth. However, as a developing country, the rapid movement of Malaysia towards industrialization makes the manufacturing sector a significant source of environmental issues within the country [50], which implies the importance of improving the environmental performance of the manufacturing sector.

Since the food industry does not cover all operational areas of green supply chain initiatives, this industry is excluded from the scope of study. For example, the initiative of eco-product design and the initiative of greening post-use in the food industry would be limited to packaging and is not applicable to food products.

ISO 14001 certified companies were selected as they are more likely to incorporate the green supply chain initiatives into their business. This is supported by previous studies [51–54].

The unit of analysis in this study is the individual company. The sampling frame was obtained from SIRIM and the Federation of Malaysian Manufacturers (FMM) directory [55] of Malaysian manufacturers.

Table 1 shows the operationalisation of the constructs that were the basis of our questionnaire development.

**Table 1:** Constructs operationalisation.

**Cluster 1: Institutional Pressures (IP)**	
**Regulatory Pressures (RIP)**	The coercive isomorphic pressures imposed by regulatory bodies to force organisations to adopt environmental strategies
**Market Pressures (MIP)**	The normative isomorphic pressures imposed by companies’ customers and export markets on companies to adopt environmental strategies
**Competitor Pressures (CIP)**	The mimic isomorphic pressures from competitors that drive companies to adopt environmental strategies
**Society Pressures (SIP)**	The coercive isomorphic pressures imposed by local communities and environmental interest groups that influence companies’ decisions on adopting environmental strategies
**Cluster 2: Firm’s Desired Competitive Advantages (CA)**	
**Cost Reduction (CRA)**	The advantage of cost reduction through making a set of functional policies and managerial attention to cost control in the firm’s value chain without ignoring the quality of goods and services
**Reputation and Legitimacy (RLA)**	The advantage of strengthening the brand and corporate image through collaboration with the firm’s key stakeholders and gaining competitive pre-emption through establishing rules, regulations, or standards that are uniquely tailored to the firm's capability
**Future Positioning (FPA)**	The advantages of gaining future market share and capturing the opportunity share by shaping the future and building new spaces in the market.
**Cluster 3: Firm’s Key Resources (KR)**	
**Continuous Improvement (CIR)**	The ability to continuously improve the firm’s processes
**Stakeholder Integration (SIR)**	The ability to integrate the views of the key stakeholders into the business processes
**Disruptive Change (DCR)**	The ability to address areas of knowledge that are uncertain, constantly evolving, and dynamically complex
**Cluster 4: Green Strategies (GS)**	
**Pollution Prevention (PPS)**	Reducing waste and emissions from the company’s current operations through incremental improvement of the company’s existing products and processes
**Product Stewardship (PSS)**	Reducing the environmental impact of a company’s existing products and processes at every stage of a product’s life cycle (from supplying raw material, though the production processes, to product consumption and disposal of end-of-life products)
**Clean Technology (CTS)**	Applying innovative clean technologies and making a disruptive change in the product and process design and gaining benefits from future market opportunities
**Cluster 5: Green Supply Chain Initiatives (GCSIs)**	
**Product design for the environment (EDP)**	Involves initiatives relating to the design of products for reuse, recycling, or recovery, design of products for reducing emissions and other environmental design objectives
**Greening Upstream (GUM)**	Relates to using environmentally friendly materials, collaboration with suppliers in environmental objectives, and other upstream activities
**Greening Production (GPN)**	Includes activities such as the optimisation of manufacturing processes to reduce waste, the consumption of materials and energy and the recycling of materials internally in the company
**Greening Downstream (GDM)**	Addresses environmentally friendly transportation, green packaging, and cooperation with customers in environmental objectives
**Greening post-Use (GPU)**	Refers to initiatives treating used products such as recovery activities for used or defective products/components or Recycling from End-of-Life products.

The dataset is comprised of 139 completed questionnaires out of 430 questionnaires that we distributed to each firm’s Environmental Management Representative (ERM). The ERM is a key informant in EMS ISO 14001-certified companies who has knowledge about green issues [56]. The given sample size of 139 companies meets the requirement of 10 times rule that implies minimum sample size should 10 times the maximum number of arrowheads pointing at a latent variable anywhere in the PLS path method [14]. Table 2 shows the profile of the respondent companies.

**Table 2:** Profile of responding companies.

Variable	Categories	Frequency	%
Type of Industry	Automotive and other Transport Equipment	25	17.99%
	Electrical and Electronics	50	35.97%
	Metal, Machinery, Equipment and Appliances	20	14.39%
	Rubber and Plastic Products	15	10.79%
	Chemical and Chemical Products	13	9.35%
	Textiles, Paper Products, Furniture, and Products of Wood	16	11.51%
Company’s age	< = 15 Years	17	12.23%
	15 Years >	122	87.77%
Company’s size	5–50	25	17.99%
	51–150	50	35.97%
	151–500	20	14.39%
	501–1000	15	10.79%
	1000>	13	9.35%
Ownership	Local owned (Fully Malaysian)	48	34.53%
	Local and Foreign Joint Venture	19	13.67%
	Foreign-based Company	72	51.80%
Market	Local	25	17.99%
	Regional/Asian	50	35.97%
	Global	20	14.39%
	Local & Regional	15	10.79%
	Local & Global	13	9.35%
Suppliers	Local	25	17.99%
	Regional/Asian	50	35.97%
	Global	20	14.39%
	Local & Regional	15	10.79%
	Local & Global	13	9.35%

We used a web-based survey solution, SurveyMonkey [57], to distribute our questionnaires and followed-up with phone call to increase the response rate. As the collected information from ERM is about the green strategies, practices, and performance of the company, this study does not constitute “human subject research”. However, regarding the confidentiality issue, we submitted a cover page with the questionnaire stating that “*the results and contributions will be solely used for academic research purposes and research article publications and no attempt will be made at identifying the individuals and/or the organizations they represent in any publications*”. We asked the respondents to fill up the questionnaire if they agree to these terms and conditions. Before analysing data, all identifiable information was removed from the data set to be insured that the respondent and its organization’s identification is kept confidential.

The collected data have been analysed by applying partial least square-based structural equation modelling (PLS-SEM) [14] using the SmartPLS software version 2 [58].

Structural equation modelling (SEM) is a second-generation statistical method that has several advantages in comparison with traditional methods such as multiple regression analysis. One of the most important feature of SEM is its ability to simultaneously test the relationships between the multiple variables as a structural model and the relationship between a latent variable and its indicators as a measurement model [14]. Recent research on green supply chains increasingly uses structural equation modelling to examine the causal relationship between green initiatives and their performance [26, 48, 59, 60].

There are two types of SEM, namely, covariance-based SEM (CB-SEM), and partial least square-based SEM (PLS_SEM). CB-SEM is applied in confirmatory research to test the established theories, while PLS-SEM is used in exploratory research to develop theories [14].

There are several reasons why researchers in the strategic management discipline use PLS-SEM. The main reasons are the ability to predict and explain the variance of key target constructs, handle the small sample size, manage the non-normal data and analyse the formatively measured constructs [61]. We have also adopted this method for two main reasons. First, because PLS-SEM focuses on prediction, we could use this method for obtaining the importance of the decision factors in achieving the key target constructs, which in our research are the green strategies adoption. Second, the ability of this method to analyse the formatively measured constructs provides us with the opportunity to calculate the weights of the measures for every GSCI. Therefore, we could develop a performance measurement system for calculating the performance of GSCIs based on the results of our empirical study. Using these features of the PLS-SEM method, we were able to conduct importance-performance matrix analysis (IPMA) [14] and obtain the ultimate priorities of GSCIs.

With regards to the factors and their relationship in the validated model, a decision-making tool was designed by using the ANP technique [13].

ANP is a more general form of the analytic hierarchy process (AHP) technique that was developed to prioritise the alternatives in a decision problem by formulating the problem as a hierarchic structures consisting of a goal, criteria, and alternatives [62]. As opposed to the hierarchic structure, ANP formulates the decision problem as a network consisting of clusters and elements within these clusters. The representation of the decision problem as a network structure allows us to see the mutual relationship between the decision clusters or elements among the different levels and the two-way relationship between the elements at the same level, indicating the inner dependencies between the elements within a cluster [13].

ANP was selected, firstly, because it is the most commonly applied method for evaluation GSCM decision alternatives. Secondly, with regards to the nature of the decision model developed in this research, which underlines the satisfaction of the requirements imposed by both external and internal drivers, simultaneously, adopting a win-win approach in the decision making procedure is critical. As Seuring [12] noted, while most model-based techniques take a trade-off approach to optimise sustainability-related decisions, the analytic network process takes a win-win approach to satisfy the decision criteria. Finally, given the complexity of the decision environment in this study, ANP is a suitable technique that can handle several decision variables included in various clusters having inner dependencies between the variables within a cluster [13]. The ability of the ANP technique to handle the complex decision environment in the research areas of environmental and supply chain management has been confirmed by several researchers [10, 11, 37, 63–65].

### Obtaining the relative intensities of clusters and elements

To obtain the relative intensities of the clusters and elements in the model, we applied two approaches. First, we made twelve sets of pairwise comparisons for determining the relative importance of the cluster’s institutional pressures, key resources, and competitive advantages and their elements in respect to the goal. Based on the AHP approach [62], we used a scale from 1 (equally important) to 9 (extremely more important) to make the pairwise comparison. For this purpose, a firm’s environmental manager representative (EMR) was asked a number of pairwise comparison questions. The environmental management representatives (EMR), who were requested to complete the pairwise comparisons questionnaire in this research, were introduced in the literature as a key informant person in the area of green supply chain management [56]. S1, S2, S3 and S4 Appendices present the questionnaires for pairwise comparisons. One example question is, “How much more important is satisfying the requirements of regulatory pressures than the market pressures while planning green strategies and initiatives?” To ensure the consistency of the judgments in making pairwise comparisons, we have also calculated the consistency ratio (C.R), which should be approximately 0.10 or less. The formula for C.R calculation can be found in most AHP and ANP books (see [66]).

Second, we prepared four matrices of relative importance by calculating the results of the total effect of a given variable on a target variable in the structural model. The total effect has been derived from a PLS path model by estimating the direct, indirect and total relationship in the structural model.

### Determining the priorities

For performing computations and obtaining the priorities in the ANP model, we used the “Super decision” software. We used the direct mode in the “Super decision” software to input the weights that were calculated based on the total effects derived from PLS path model. After entering the raw data in the “Super decision” software, the computations were performed, forming the unweighted supermatrix, weighted supermatrix, and limit matrix. The limit matrix provides the relative priorities for each alternative considered within the decision framework. These priorities show the importance of every green strategy in satisfying the internal and external factors and the importance of every GSCI in achieving the prioritised green strategies. To ensure the model stability, we executed sensitivity analysis, which shows how the optimal solution responds to changes in input parameters.

To adjust the priorities according to the current performance of the company, it was necessary to calculate the company’s performance in every area of green supply chain operation and conduct the importance-performance analysis. This technique can improve the method of prioritisation [67].

To conduct an importance-performance analysis, we developed a performance measurement system including the measurement indicators and their weights associated with the GSCIs. The indicators are the formative measures that we have already collected data for and analysed in the PLS-SEM. The weights for these formative measures are also derived from the results of measurement model in the PLS-SEM. To calculating the performance, we asked each company to assign a score between 0 (not at all) to 100 (excellent) to each indicator. The final priorities of GSCIs were made based on importance-performance matrix analysis (IPMA).

### Evaluation of the method’s usability

As the last phase of the research, the usability of the developed prioritisation method was evaluated using the case study. To evaluate the method’s usability, a 2-hour feedback session was conducted by involving the environmental management representative (EMR) in the process of method’s implementation and evaluation.

For conducting a systematic evaluation, an interview protocol was also developed to consider the user’s feedback through semi-structured interviews. This interview protocol included questions on usability dimensions, namely, learnability, efficiency, and helpfulness, which were selected from the dimensions suggested by the literature for evaluation of the software usability [68–70]. These three dimensions were selected because they were frequently addressed in various usability questionnaires and were also more relevant to the evaluation purpose of this research.

At the beginning of the meeting, the purpose of the method, the concept behind the prioritisation method, the procedure for prioritising green strategies and green supply chain initiatives, and the main functions of the method were briefly explained for the contributors in the evaluation process. The EMR was then asked to provide the input regarding the current performance score of the green supply chain initiatives in his/her company. After inputting the performance scores, the EMR was asked to make pairwise comparisons. By taking these inputs, the final report showing the priority vector of green supply chain initiatives was made using the Super Decisions [71] software, which is a free software for ANP and AHP models, and an Excel sheet which was designed for analyzing the importance-performance. At the end of the feedback session, the EMR was asked to express her opinion about the method’s usability in terms of learnability, efficiency and helpfulness. These feedback was then summarized and analysed.

## Establishing the Decision Model

**Fig 2:**
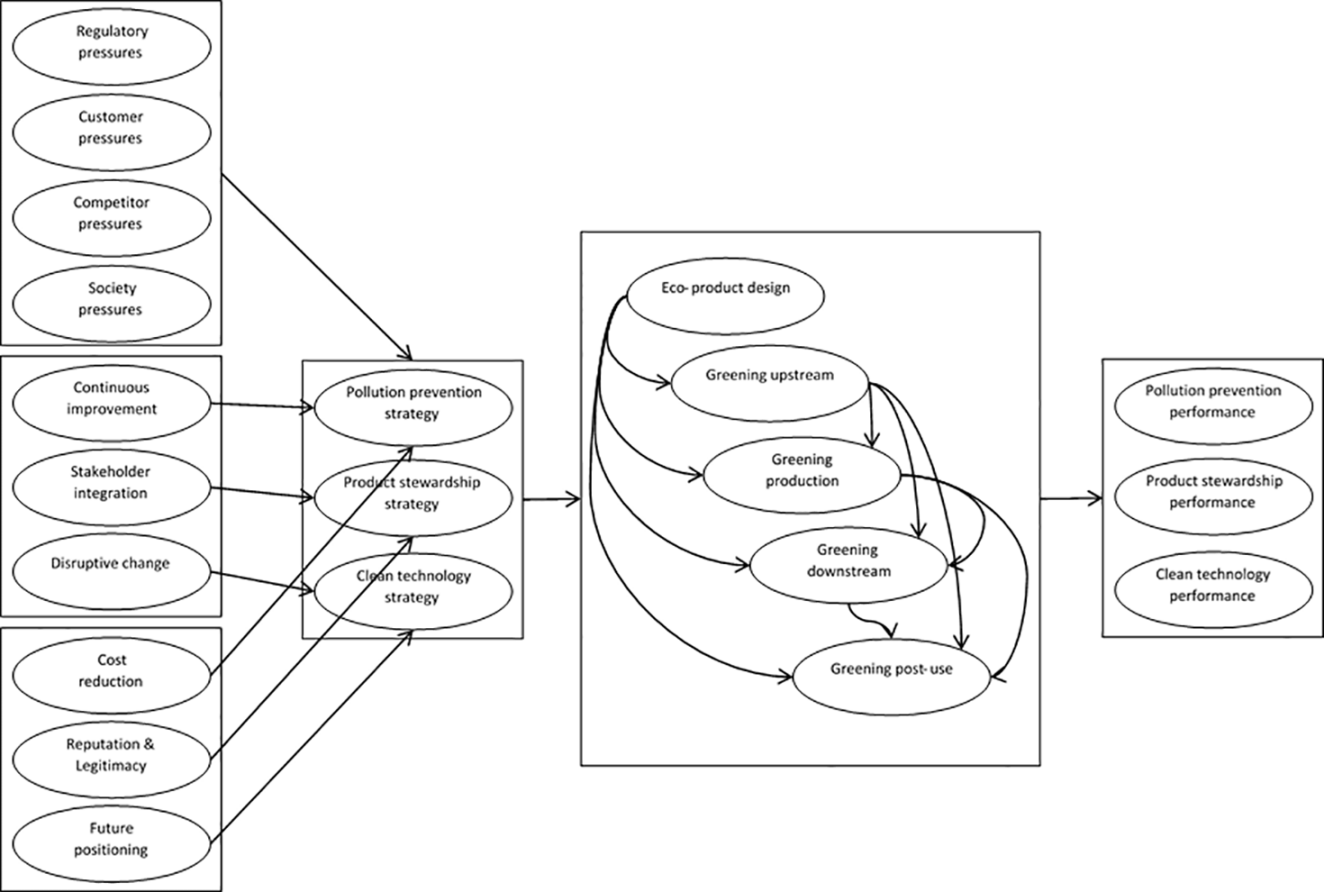
Causal relationship between the decision elements.

According to the findings from the literature review (presented in section 2), we have introduced five clusters in our decision model, namely, institutional pressures, competitive advantages, key resources, environmental strategies, and GSCIs. Each of these clusters has its own elements, as shown in Table 1. In order investigate the relationship between these elements, we have developed a causal relationship model and tested the model with a dataset generated from the survey amongst the ISO 14001-certified manufacturers in Malaysia. Fig 2 shows the conceptual model.

To test the conceptual model we applied the partial l square-based SEM (PLS-SEM) technique. To ensure the validity and reliability of the model, we evaluated the measurement model for both reflective and formative measures.

### Validity and Reliability

To evaluate the reflective measurement model, we analysed composite reliability (CR), convergent validity, and discriminate validity following the methodology documented by Hair et al. [14]. Table 3 depicts the results of the reflective measurement model tests. The factor loadings for all reflective items (except the item RIP1, which was deleted) exceeded the recommended value of 0.7. Composite reliability, which is a measure of internal consistency reliability, ranged from 0.815 to 0.946, which is acceptable [72]. Convergent validity refers to the extent to which a measure positively correlates with alternative measures of the same construct [14]. To analyse the convergent validity, we considered the outer loadings of the indicators and the average variance extracted (AVE). All the values of AVE, which refers to the communality of the constructs [14], are acceptable according to the minimum recommended value of 0.5 [73, 74].

**Table 3:** Evaluation of reflective measurement model.

Construct	Item	Loading	AVE	CR
**Regulatory pressures**	RIP2	0.619	0.526	0.815
	RIP3	0.731		
	RIP4	0.787		
	RIP5	0.754		
**Customer pressures**	MIP1	0.741	0.627	0.909
	MIP2	0.743		
	MIP3	0.744		
	MIP4	0.807		
	MIP5	0.867		
	MIP6	0.838		
**Competitor pressures**	CIP1	0.768	0.715	0.937
	CIP2	0.865		
	CIP3	0.876		
	CIP4	0.861		
	CIP5	0.816		
	CIP6	0.881		
**Society pressures**	SIP1	0.719	0.507	0.837
	SIP2	0.764		
	SIP3	0.704		
	SIP4	0.653		
	SIP5	0.716		
**Cost reduction**	CRA1	0.922	0.791	0.919
	CRA2	0.909		
	CRA3	0.835		
**Reputation and legitimacy**	RLA1	0.828	0.635	0.874
	RLA2	0.817		
	RLA3	0.752		
	RLA4	0.789		
**Future positioning**	FPA1	0.831	0.773	0.931
	FPA2	0.866		
	FPA3	0.903		
	FPA4	0.914		
**Continuous improvement**	CIR1	0.764	0.699	0.921
	CIR2	0.793		
	CIR3	0.880		
	CIR4	0.860		
	CIR5	0.878		
**Stakeholder integration**	SIR1	0.883	0.757	0.949
	SIR2	0.921		
	SIR3	0.923		
	SIR4	0.896		
	SIR5	0.734		
	SIR6	0.851		
**Disruptive change**	DCR1	0.814	0.736	0.933
	DCR2	0.874		
	DCR3	0.886		
	DCR4	0.823		
	DCR5	0.889		
**Pollution prevention strategy**	PPS1	0.931	0.846	0.943
	PPS2	0.944		
	PPS3	0.884		
**Product stewardship strategy**	PSS1	0.880	0.805	0.943
	PSS2	0.915		
	PSS3	0.894		
	PSS4	0.900		
**Clean technology strategy**	CTS1	0.878	0.814	0.946
	CTS2	0.929		
	CTS3	0.884		
	CTS4	0.918		

Discriminate validity refers to the distinction of a construct from the other constructs in the measurement model. To test the discriminate validity, we used Fronell-Larcker criterion approach. According to this approach, the square root of the AVE for each construct must be larger than its correlation with other constructs [14]. This held true for our measurement model (see Table 4).

**Table 4:** Fronell-Larcker criterion analysis.

	RIP	MIP	CIP	SIP	CRA	RLA	FPA	CIR	SIR	DCR	PPS	PSS	CTS	EDP	GUM	GPN	GDM	GPU	ENpp	ENps	ENct
**RIP**	**0.725**																				
**MIP**	0.393	**0.792**																			
**CIP**	0.405	0.503	**0.845**																		
**SIP**	0.279	0.367	0.313	**0.712**																	
**CRA**	0.204	0.174	0.411	0.217	**0.890**																
**RLA**	0.357	0.217	0.583	0.288	0.518	**0.797**															
**FPA**	0.231	0.173	0.566	0.208	0.502	0.736	**0.879**														
**CIR**	0.403	0.218	0.493	0.263	0.470	0.508	0.532	**0.836**													
**SIR**	0.427	0.310	0.564	0.281	0.362	0.502	0.488	0.638	**0.870**												
**DCR**	0.368	0.226	0.533	0.302	0.324	0.618	0.551	0.654	0.756	**0.858**											
**PPS**	0.380	0.253	0.524	0.255	0.434	0.545	0.568	0.586	0.468	0.511	**0.920**										
**PSS**	0.399	0.402	0.585	0.318	0.419	0.612	0.540	0.567	0.595	0.637	0.628	**0.897**									
**CTS**	0.331	0.323	0.586	0.301	0.417	0.592	0.635	0.566	0.545	0.647	0.674	0.757	**0.902**								
**EDP**	0.254	0.273	0.362	0.315	0.265	0.350	0.399	0.343	0.307	0.408	0.534	0.505	0.572	**NA**							
**GUM**	0.327	0.426	0.500	0.285	0.280	0.346	0.344	0.430	0.457	0.505	0.523	0.606	0.687	0.710	**NA**						
**GPN**	0.326	0.336	0.425	0.335	0.287	0.306	0.346	0.335	0.364	0.397	0.566	0.497	0.618	0.699	0.801	**NA**					
**GDM**	0.302	0.442	0.496	0.437	0.249	0.389	0.344	0.401	0.450	0.476	0.425	0.554	0.660	0.614	0.747	0.665	**NA**				
**GPU**	0.232	0.390	0.308	0.201	0.042	0.226	0.147	0.163	0.299	0.328	0.201	0.324	0.398	0.358	0.557	0.531	0.519	**NA**			
**ENpp**	0.272	0.247	0.420	0.300	0.228	0.360	0.422	0.441	0.346	0.389	0.545	0.494	0.609	0.586	0.666	0.678	0.620	0.474	**NA**		
**ENps**	0.229	0.256	0.316	0.339	0.094	0.301	0.226	0.239	0.285	0.332	0.318	0.384	0.467	0.463	0.596	0.655	0.649	0.630	0.696	**NA**	
**ENct**	0.307	0.333	0.338	0.307	0.190	0.320	0.320	0.341	0.300	0.367	0.497	0.474	0.615	0.629	0.710	0.735	0.641	0.542	0.838	0.753	**NA**

To evaluate the formative measurement model, we first assessed the collinearity issues by examining the variance inflation factors (VIF), which are recommended to be less than the value of 5 [14]. Except for one indicator (GDMcm2), all the VIF in our formative measurement model satisfy this condition (see Table 5). However, because this item is not interchangeable and it satisfies the condition of VIF less than 10 recommended by the general statistics theory [75, 76], we retain this variable. Second, we proceeded with analysing the significance of the outer weights. For this purpose, we ran the bootstrapping procedure by creating 1000 random subsamples. The t-value calculated after running this procedure shows each indicator weight’s significance. As seen from Table 5, there are several indicators that have an insignificant weight (less than 1.645). However, we did not delete these items because their outer loadings are above 0.5, which shows that the item has absolute importance but no relative importance [14].

**Table 5:** Evaluation of formative measurement model.

Construct		Item	Weight	Loading	t-value	VIF
**Product design for**		EDP1	0.070	0.795	0.383	2.539
**the environment**		EDP2	0.230	0.860	1.615	2.631
		EDP3	0.286	0.889	1.694	3.157
		EDP4	0.383	0.929	2.257	3.640
		EDP5	0.154	0.887	0.913	3.690
**Greening**	Green purchasing	GUMpur1	0.297	0.750	2.618	1.514
**upstream**		GUMpur2	0.269	0.865	2.061	2.420
		GUMpur3	0.583	0.935	5.211	2.263
	Green supplier management	GUMsm1	0.373	0.868	2.480	2.246
		GUMsm2	0.344	0.908	2.118	3.268
		GUMsm3	0.282	0.905	1.415	4.637
		GUMsm4	0.125	0.880	0.734	4.051
**Green production**		GPN1	0.217	0.766	1.844	2.555
		GPN2	0.119	0.758	0.738	2.495
		GPN3	0.001	0.744	0.005	3.062
		GPN4	0.209	0.781	1.313	3.690
		GPN5	0.299	0.827	2.371	2.276
		GPN6	0.266	0.809	2.132	2.148
		GPN7	0.183	0.640	1.417	1.455
**Greening**	Green distribution	GDM1dis	0.102	0.699	0.899	1.749
**downstream**		GDM2dis	0.562	0.935	3.016	2.367
		GDM3dis	0.455	0.887	3.080	1.876
	Green customer management	GDMcm1	0.390	0.849	2.989	1.916
		GDMcm2	0.044	0.853	0.157	5.410
		GDMcm3	0.138	0.824	0.930	4.012
		GDMcm4	0.073	0.828	0.309	4.201
		GDMcm5	0.166	0.823	0.908	3.942
		GDMcm6	0.349	0.919	1.693	3.360
**Greening**	Packaging recovery	GPUpack1	0.219	0.630	1.213	1.353
**post-use**		GPUpack2	0.629	0.904	4.135	1.431
		GPUpack3	0.363	0.809	1.989	1.641
	Product recovery	GPUpro1	0.109	0.646	0.571	1.557
		GPUpro2	0.333	0.745	1.795	1.479
		GPUpro3	0.440	0.831	2.715	1.496
		GPUpro4	0.392	0.805	2.486	1.593
	Investment recovery	GPUirec1	0.469	0.834	2.558	1.645
		GPUirec2	0.432	0.826	2.417	1.687
		GPUirec3	0.200	0.578	0.777	3.044
		GPUirec4	0.222	0.619	0.914	3.137
**Strategic**	Pollution prevention performance	ENPpp1	0.437	0.794	2.532	1.548
**environmental**		ENPpp2	0.348	0.828	1.977	2.293
**performance**		ENPpp3	0.139	0.780	0.760	2.409
		ENPpp4	0.380	0.674	2.464	1.198
	Product stewardship performance	ENPps1	0.388	0.768	1.906	1.647
		ENPps2	0.244	0.767	1.259	2.036
		ENPps3	0.122	0.824	0.529	3.108
		ENPps4	0.139	0.734	0.781	2.735
		ENPps5	-0.070	0.795	0.314	4.052
		ENPps6	0.441	0.834	2.440	3.259
	Clean technology performance	ENPct1	0.199	0.781	1.477	2.412
		ENPct2	0.235	0.787	1.312	3.676
		ENPct3	0.117	0.770	0.684	3.489
		ENPct4	0.421	0.811	2.804	1.521
		ENPct5	0.287	0.794	1.858	1.719

Common method variance (CMV) has been also examined by using Harmen’s single factor test in the SPSS software and the results showed that the measures are not affected by CMB.

### Significant causal relationships

**Fig 3:**
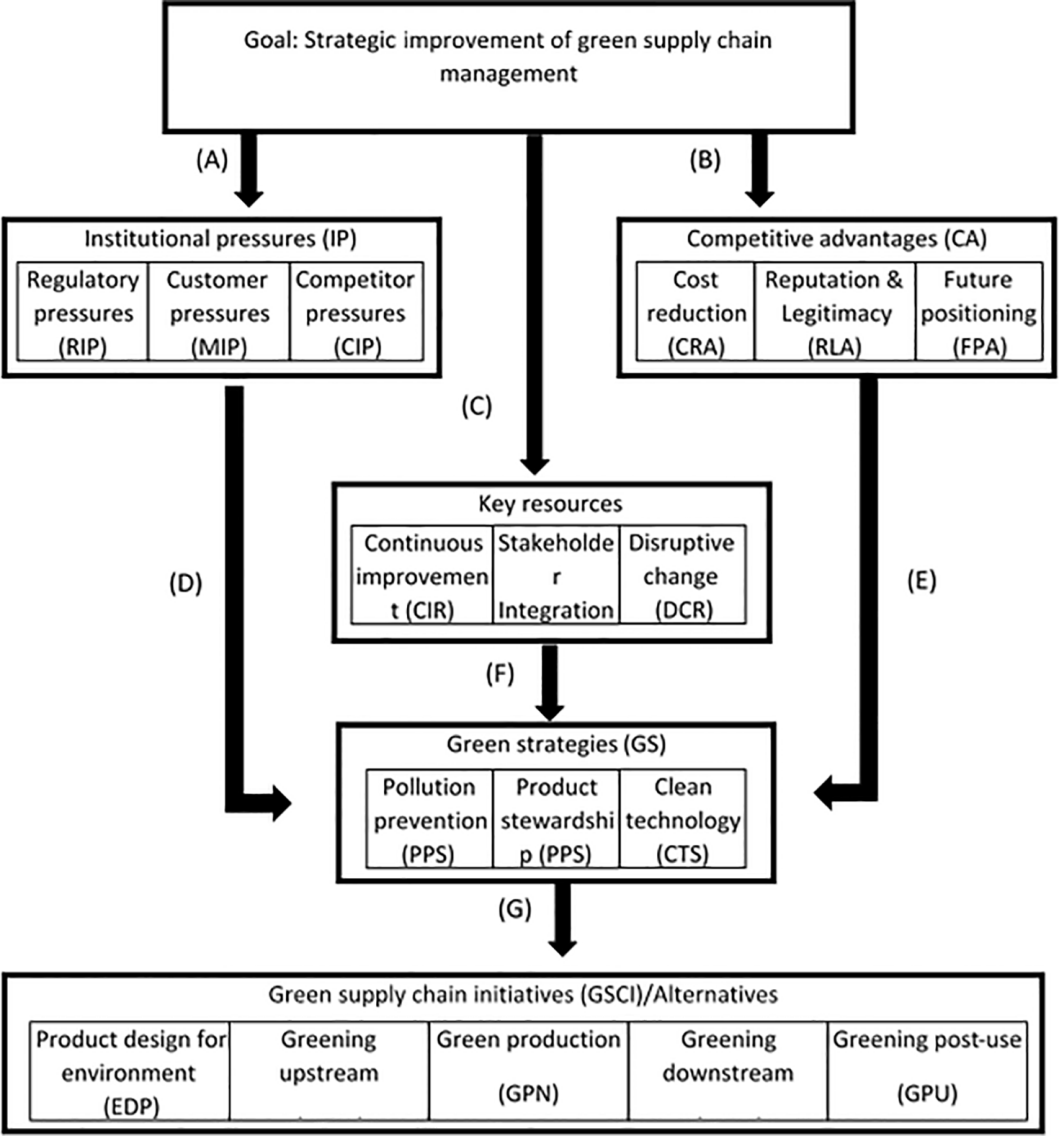
Decision model for improvement of strategic green supply chain management.

To investigate how each decision element in a cluster influences its related elements in the other clusters, we evaluated the structural model to investigate the proposed relationship in our conceptual model. Table 6 shows the results presenting the significant and non-significant relationships. The decision model was formed based on the significant relationships. For example, according to the results, the element of society pressures has no significant relationship with the elements in the cluster green strategy, so we removed this element from our final decision model (see Fig 3).

**Table 6:** Evaluation of structural model.

Relation		Path coefficient	Standard Error	T-Value	Result
**RIP -> PPS**	Regulatory pressures->Pollution prevention strategy	0.121	0.075	1.617*	Significant
**RIP -> PSS**	Regulatory pressures->Product stewardship strategy	0.036	0.060	0.606	Non-significant
**RIP -> CTS**	Regulatory pressures->Clean technology strategy	0.015	0.068	0.224	Non-significant
**MIP -> PPS**	Customer pressures->Pollution prevention strategy	-0.035	0.092	0.382	Non-significant
**MIP -> PSS**	Customer pressures->Product stewardship strategy	0.122	0.079	1.548*	Significant
**MIP -> CTS**	Customer pressures->Clean technology strategy	0.083	0.084	0.995	Non-significant
**CIP -> PPS**	Competitor pressures->Pollution prevention strategy	0.263	0.079	3.344***	Significant
**CIP -> PSS**	Competitor pressures->Product stewardship strategy	0.143	0.089	1.612*	Significant
**CIP -> CTS**	Competitor pressures->Clean technology strategy	0.149	0.086	1.728**	Significant
**SIP -> PPS**	Society pressures->Pollution prevention strategy	0.033	0.077	0.422	Non-significant
**SIP -> PSS**	Society pressures->Product stewardship strategy	0.045	0.073	0.612	Non-significant
**SIP -> CTS**	Society pressures->Clean technology strategy	0.052	0.066	0.784	Non-significant
**CRA -> PPS**	Cost reduction->Pollution prevention strategy	0.135	0.074	1.831**	Significant
**RLA -> PSS**	Reputation and legitimacy ->Product stewardship strategy	0.334	0.092	3.644***	Significant
**FPA -> CTS**	Future positioning->Clean technology strategy	0.332	0.081	4.115***	Significant
**CIR -> PPS**	Continuous improvement->Pollution prevention strategy	0.336	0.096	3.510***	Significant
**SIR -> PSS**	Stakeholder integration ->Product stewardship strategy	0.284	0.075	3.762***	Significant
**DCR -> CTS**	Disruptive change->Clean technology strategy	0.347	0.078	4.477***	Significant
**PPS -> EDP**	Pollution prevention strategy->Product design for the environment	0.251	0.126	1.989**	Significant
**PPS -> GUM**	Pollution prevention strategy->Greening upstream	-0.042	0.073	0.581	Non-significant
**PPS -> GPN**	Pollution prevention strategy->Green production	0.180	0.081	2.220**	Significant
**PPS -> GDM**	Pollution prevention strategy->Greening downstream	-0.194	0.082	2.366***	Significant
**PPS -> GPU**	Pollution prevention strategy->Greening post-use	-0.222	0.117	1.902**	Significant
**PSS -> EDP**	Product stewardship strategy->Product design for the environment	0.096	0.119	0.805	Non-significant
**PSS -> GUM**	Product stewardship strategy->Greening upstream	0.151	0.080	1.902**	Significant
**PSS -> GPN**	Product stewardship strategy->Green production	-0.146	0.075	1.942**	Significant
**PSS -> GDM**	Product stewardship strategy->Greening downstream	0.071	0.110	0.646	Non-significant
**PSS -> GPU**	Product stewardship strategy->Greening post-use	0.051	0.134	0.384	Non-significant
**CTS -> EDP**	Clean technology strategy-Product design for the environment	0.330	0.138	2.391***	Significant
**CTS -> GUM**	Clean technology strategy->Greening upstream	0.333	0.101	3.310***	Significant
**CTS -> GPN**	Clean technology strategy->Green production	0.090	0.100	0.903	Non-significant
**CTS -> GDM**	Clean technology strategy->Greening downstream	0.303	0.119	2.540***	Significant
**CTS -> GPU**	Clean technology strategy->Greening post-use	0.060	0.149	0.402	Non-significant
**EDP -> GUM**	Product design for the environment->Greening upstream	0.468	0.082	5.692***	Significant
**EDP -> GPN**	Product design for the environment->Green production	0.219	0.087	2.530***	Significant
**EDP -> GDM**	Product design for the environment->Greening downstream	0.118	0.091	1.302*	Significant
**EDP -> GPU**	Product design for the environment->Greening post-use	-0.164	0.128	1.286*	Significant
**GUM -> GPN**	Greening upstream->Green production	0.569	0.089	6.424***	Significant
**GUM -> GDM**	Greening upstream->Greening downstream	0.410	0.119	3.459***	Significant
**GUM -> GPU**	Greening upstream->Greening post-use	0.190	0.186	1.022	Non-significant
**GPN -> GDM**	Green production-Greening downstream	0.137	0.089	1.534*	Significant
**GPN -> GPU**	Green production->Greening post-use	0.370	0.131	2.825***	Significant
**GDM -> GPU**	Greening downstream->Greening post-use	0.240	0.145	1.658**	Significant
**EDP -> ENpp**	Product design for the environment->Pollution prevention performance	0.125	0.119	1.044	Non-significant
**EDP -> ENps**	Product design for the environment->Product stewardship performance	-0.021	0.088	0.245	Non-significant
**EDP -> ENct**	Product design for the environment->Clean technology performance	0.140	0.078	1.788**	Significant
**GUM -> ENpp**	Greening upstream->Pollution prevention performance	0.159	0.120	1.322*	Significant
**GUM -> ENps**	Greening upstream->Product stewardship performance	0.014	0.103	0.141	Non-significant
**GUM -> ENct**	Greening upstream->Clean technology performance	0.160	0.111	1.449*	Significant
**GPN -> ENpp**	Green production->Pollution prevention performance	0.327	0.142	2.303**	Significant
**GPN -> ENps**	Green production->Product stewardship performance	0.296	0.123	2.399***	Significant
**GPN -> ENct**	Green production->Clean technology performance	0.327	0.122	2.689***	Significant
**GDM -> ENpp**	Greening downstream->Pollution prevention performance	0.190	0.094	2.034**	Significant
**GDM -> ENps**	Greening downstream->Product stewardship performance	0.313	0.102	3.078***	Significant
**GDM -> ENct**	Greening downstream->Clean technology performance	0.143	0.080	1.782**	Significant
**GPU -> ENpp**	Greening post-use->Pollution prevention performance	0.054	0.084	0.638	Non-significant
**GPU -> ENps**	Greening post-use->Product stewardship performance	0.306	0.084	3.647***	Significant
**GPU -> ENct**	Greening post-use->Clean technology performance	0.152	0.077	1.978**	Significant

*p<0.1

**p<0.05

***p<0.01

## Obtaining the Relative Intensities of Clusters and Elements

### Results from the case study

To obtain the relative intensities of the first three clusters and their elements with respect to the objective of the strategic improvement of green supply chain management, we suggest using the firm’s environmental management representative’s judgment for making pairwise comparison between the clusters and elements. The reason behind this is that the environmental management representative is a key informant in Malaysian ISO 14001-certified companies who has the appropriate knowledge about green issues [56]. We have already executed this pairwise comparison in company X as our case study in this research.

Company X is a subsidiary of a Japanese manufacturer that produces electronic components in Malaysia. The company is a large-size company with more than 15 years experiences in the electronics manufacturing industry. The company works with several suppliers from Asia, and its products are sold in Southeast Asia and the rest of the world.

Similar to AHP, pairwise comparison is made based on a scale from 1 to 9 in which 1 indicates the equal importance of two elements regarding their contribution to the objective and 9 indicates the absolute importance of one element over the other.

The responses to pairwise comparisons are presented in S5, S6, S7, and S8 Appendices to show the calculation of the relative weights for the clusters and their associated elements in the first level of the decision model. The calculation is made by using the Super Decisions [71] software, which is a free software for ANP and AHP models.

The relative weights of the clusters with respect to the goal in company X are 0.121957, 0.558425, and 0.319618 for institutional pressures, competitive advantages, and key resources, respectively.

### 5.2 Results from the survey

To obtain the relative intensities of the elements in the cluster green strategies and GSCIs, we calculated the total effect of each independent element on its associated dependent variables. For this purpose, we ran the PLS algorithm for the modified causal relationship model including only significant relationships. The results of the total effect are shown in Table 7.

**Table 7:** The total effect of the elements on the target variable

	Pollution prevention	Product stewardship	Clean technology
**Regulatory pressures**	0.1191	0.0000	0.0000
**Customer pressures**	0.0000	0.1435	0.0000
**Competitor pressures**	0.2532	0.1451	0.2143
**Cost reduction**	0.1379	0.0000	0.0000
**Reputation and legitimacy**	0.0000	0.3457	0.0000
**Future positioning**	0.0000	0.0000	0.3146
**Continuous improvement**	0.3408	0.0000	0.0000
**Stakeholder integration**	0.0000	0.2982	0.0000
**Disruptive change**	0.0000	0.0000	0.3597
**Product design for the environment**	0.3659	0.3640	0.4716
**Greening upstream**	0.5511	0.4622	0.5068
**Greening production**	0.3967	0.4783	0.4151
**Greening downstream**	0.2130	0.4105	0.1851
**Greening post-use**	0.0000	0.3148	0.1440

Then, by normalising the values of the elements in one cluster with respect to its control variable in another cluster, we obtained the relative priority vector. For example, the results of our survey show that competitor pressures has a total effect of 0.2532, 0.1451, and 0.2143 on the adoption of pollution prevention, product stewardship, and clean technology strategy, respectively. By normalising these total effects, we can obtain the relative importance of each strategy in satisfying the requirements of competitive pressures by 0.41332, 0.236859, and 0.34982, respectively.

### Determining the Priorities

#### Computing the importance

After obtaining all the relative intensities of the clusters and elements in the ANP model, the importance of green strategies and GSCIs was computed by forming the supermatrix. We used the Super Decisions [71] software to form the supermatrix, perform the computations and obtaining the limiting priorities.

Tables 8, 9, and 10 show the unweighted, weighted, and limiting supermatrix, respectively.

**Table 8:** Unweighted supermatrix.

		Goal	Institutional pressures			Competitive advantages			Key resources			Green strategies			Green supply chain initiatives				
		Goal	RIP	MIP	CIP	CRA	RLA	FPA	CIR	SIR	DCR	PPS	PSS	CTS	EDP	GUM	GPN	GDM	GPU
**Goal**	Goal	0	0	0	0	0	0	0	0	0	0	0	0	0	0	0	0	0	0
**Institutional**	RIP	0.33	0	0	0	0	0	0	0	0	0	0	0	0	0	0	0	0	0
**pressures**	MIP	0.26	0	0	0	0	0	0	0	0	0	0	0	0	0	0	0	0	0
	CIP	0.41	0	0	0	0	0	0	0	0	0	0	0	0	0	0	0	0	0
**Competitive**	CRA	0.33	0	0	0	0	0	0	0	0	0	0	0	0	0	0	0	0	0
**advantages**	RLA	0.33	0	0	0	0	0	0	0	0	0	0	0	0	0	0	0	0	0
	FPA	0.33	0	0	0	0	0	0	0	0	0	0	0	0	0	0	0	0	0
**Key**	CIR	0.33	0	0	0	0	0	0	0	0	0	0	0	0	0	0	0	0	0
**resources**	SIR	0.33	0	0	0	0	0	0	0	0	0	0	0	0	0	0	0	0	0
	DCR	0.33	0	0	0	0	0	0	0	0	0	0	0	0	0	0	0	0	0
**Green**	PPS	0	1.00	0	0.41	1.00	0	0	1.00	0	0	0	0	0	0	0	0	0	0
**strategies**	PSS	0	0	1.00	0.24	0	1.00	0	0	1.00	0	0	0	0	0	0	0	0	0
	CTS	0	0	0	0.35	0	0	1.00	0	0	1.00	0	0	0	0	0	0	0	0
**Green supply**	EDP	0	0	0	0	0	0	0	0	0	0	0.24	0.18	0.27	0	0	0	0	0
**chain**	GUM	0	0	0	0	0	0	0	0	0	0	0.36	0.23	0.29	0	0	0	0	0
**initiatives**	GPN	0	0	0	0	0	0	0	0	0	0	0.26	0.24	0.24	0	0	0	0	0
	GDM	0	0	0	0	0	0	0	0	0	0	0.14	0.20	0.11	0	0	0	0	0
	GPU	0	0	0	0	0	0	0	0	0	0	0	0.16	0.08	0	0	0	0	0

**Table 9:** Weighted supermatrix.

		Goal	Institutional pressures			Competitive advantages			Key resources			Green strategies			Green supply chain initiatives				
		Goal	RIP	MIP	CIP	CRA	RLA	FPA	CIR	SIR	DCR	PPS	PSS	CTS	EDP	GUM	GPN	GDM	GPU
**Goal**	Goal	0	0	0	0	0	0	0	0	0	0	0	0	0	0	0	0	0	0
**Institutional**	RIP	0.04	0	0	0	0	0	0	0	0	0	0	0	0	0	0	0	0	0
**pressures**	MIP	0.03	0	0	0	0	0	0	0	0	0	0	0	0	0	0	0	0	0
	CIP	0.05	0	0	0	0	0	0	0	0	0	0	0	0	0	0	0	0	0
**Competitive**	CRA	0.19	0	0	0	0	0	0	0	0	0	0	0	0	0	0	0	0	0
**advantages**	RLA	0.19	0	0	0	0	0	0	0	0	0	0	0	0	0	0	0	0	0
	FPA	0.19	0	0	0	0	0	0	0	0	0	0	0	0	0	0	0	0	0
**Key**	CIR	0.11	0	0	0	0	0	0	0	0	0	0	0	0	0	0	0	0	0
**resources**	SIR	0.11	0	0	0	0	0	0	0	0	0	0	0	0	0	0	0	0	0
	DCR	0.11	0	0	0	0	0	0	0	0	0	0	0	0	0	0	0	0	0
**Green**	PPS	0	1.00	0	0.41	1.00	0	0	1.00	0	0	0	0	0	0	0	0	0	0
**strategies**	PSS	0	0	1.00	0.24	0	1.00	0	0	1.00	0	0	0	0	0	0	0	0	0
	CTS	0	0	0	0.35	0	0	1.00	0	0	1.00	0	0	0	0	0	0	0	0
**Green supply**	EDP	0	0	0	0	0	0	0	0	0	0	0.24	0.18	0.27	0	0	0	0	0
**chain**	GUM	0	0	0	0	0	0	0	0	0	0	0.36	0.23	0.29	0	0	0	0	0
**initiatives**	GPN	0	0	0	0	0	0	0	0	0	0	0.26	0.24	0.24	0	0	0	0	0
	GDM	0	0	0	0	0	0	0	0	0	0	0.14	0.20	0.11	0	0	0	0	0
	GPU	0	0	0	0	0	0	0	0	0	0	0	0.16	0.08	0	0	0	0	0

**Table 10:** Limit supermatrix.

		Goal	Institutional pressures			Competitive advantages			Key resources			Green strategies			Green supply chain initiatives				
		Goal	RIP	MIP	CIP	CRA	RLA	FPA	CIR	SIR	DCR	PPS	PSS	CTS	EDP	GUM	GPN	GDM	GPU
**Goal**	Goal	0	0	0	0	0	0	0	0	0	0	0	0	0	0	0	0	0	0
**Institutional**	RIP	0.01	0	0	0	0	0	0	0	0	0	0	0	0	0	0	0	0	0
**pressures**	MIP	0.01	0	0	0	0	0	0	0	0	0	0	0	0	0	0	0	0	0
	CIP	0.02	0	0	0	0	0	0	0	0	0	0	0	0	0	0	0	0	0
**Competitive**	CRA	0.06	0	0	0	0	0	0	0	0	0	0	0	0	0	0	0	0	0
**advantages**	RLA	0.06	0	0	0	0	0	0	0	0	0	0	0	0	0	0	0	0	0
	FPA	0.06	0	0	0	0	0	0	0	0	0	0	0	0	0	0	0	0	0
**Key**	CIR	0.04	0	0	0	0	0	0	0	0	0	0	0	0	0	0	0	0	0
**resources**	SIR	0.04	0	0	0	0	0	0	0	0	0	0	0	0	0	0	0	0	0
	DCR	0.04	0	0	0	0	0	0	0	0	0	0	0	0	0	0	0	0	0
**Green**	PPS	0.12	0.50	0	0.21	0.50	0	0	0.50	0	0	0	0	0	0	0	0	0	0
**strategies**	PSS	0.11	0	0.50	0.12	0	0.50	0	0	0.50	0	0	0	0	0	0	0	0	0
	CTS	0.10	0	0	0.17	0	0	0.50	0	0	0.50	0	0	0	0	0	0	0	0
**Green**	EDP	0.08	0.12	0.09	0.12	0.12	0.09	0.14	0.12	0.09	0.14	0.24	0.18	0.27	0	0	0	0	0
**supply**	GUM	0.10	0.18	0.11	0.15	0.18	0.11	0.15	0.18	0.11	0.15	0.36	0.23	0.29	0	0	0	0	0
**chain**	GPN	0.08	0.13	0.12	0.12	0.13	0.12	0.12	0.13	0.12	0.12	0.26	0.24	0.24	0	0	0	0	0
**initiatives**	GDM	0.05	0.07	0.10	0.07	0.07	0.10	0.05	0.07	0.10	0.05	0.14	0.20	0.11	0	0	0	0	0
	GPU	0.03	0	0.08	0.03	0	0.08	0.04	0	0.08	0.04	0	0.16	0.08	0	0	0	0	0

The unweighted supermatrix is an arrangement of all priority vectors, representing the impact of a given set of elements in a cluster on another element in the network. The priorities vector for the elements in each cluster can be seen from the unweighted supermatrix.

The weighted supermatrix was formed by normalising the values in every column of the unweighted supermatrix, so that the sum of the column was equal to the value of 1. To normalise the values, the relative score of each element was multiplied by the relative importance of its cluster.

The optimum solution was obtained using the limiting priorities. The limiting priorities were computed by multiplying the weighted supermatrix by itself n times until the columns stabilised. The final ranking shows the influence of each of the GSCIs on the objective of the strategic improvement of green supply chain management. According to the results, the ranking of the green strategy is: 1-pollution prevention, 2-product stewardship, and 3-clean technology. For the implementation of this ranking of green strategies, the importance of each green initiative is: 1-greening upstream, 2-green production, 3-product design for the environment, 4-greening downstream, and 5-greening post-use.

**Fig 4:**
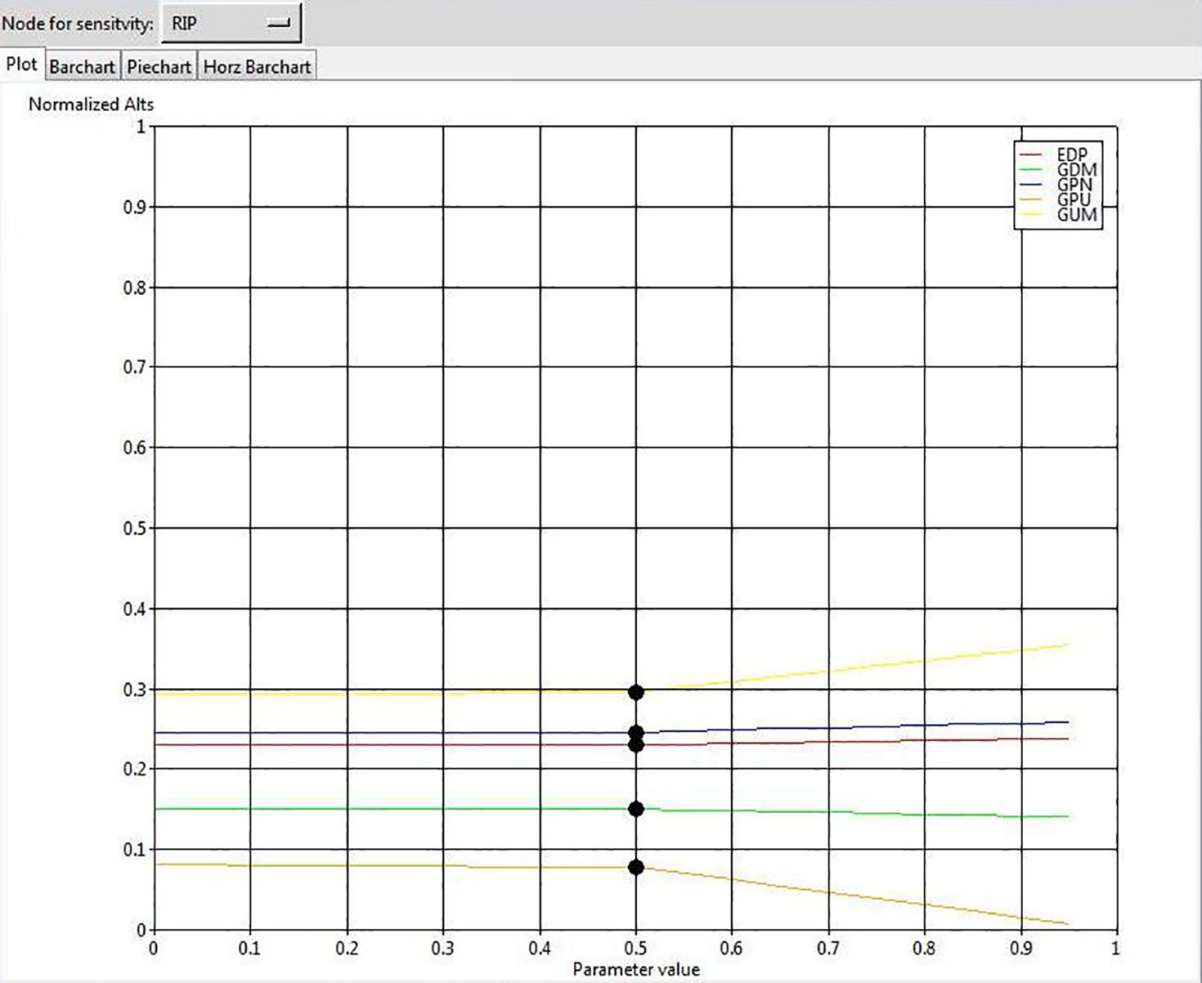
The results of sensitivity analysis for the node “Regulatory Pressures”.

**Fig 5:**
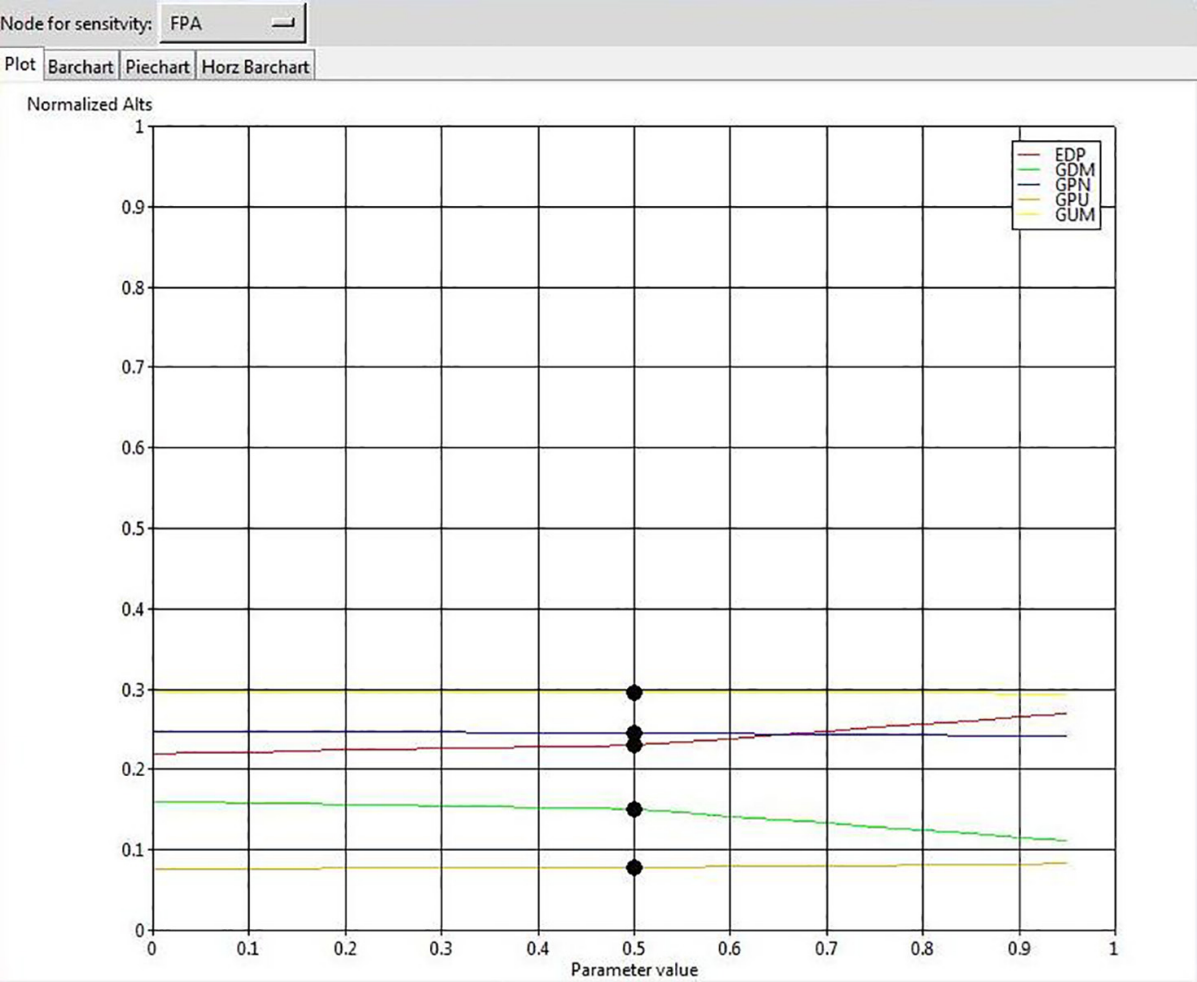
The results of sensitivity analysis for the node “Future Positioning”.

**Fig 6:**
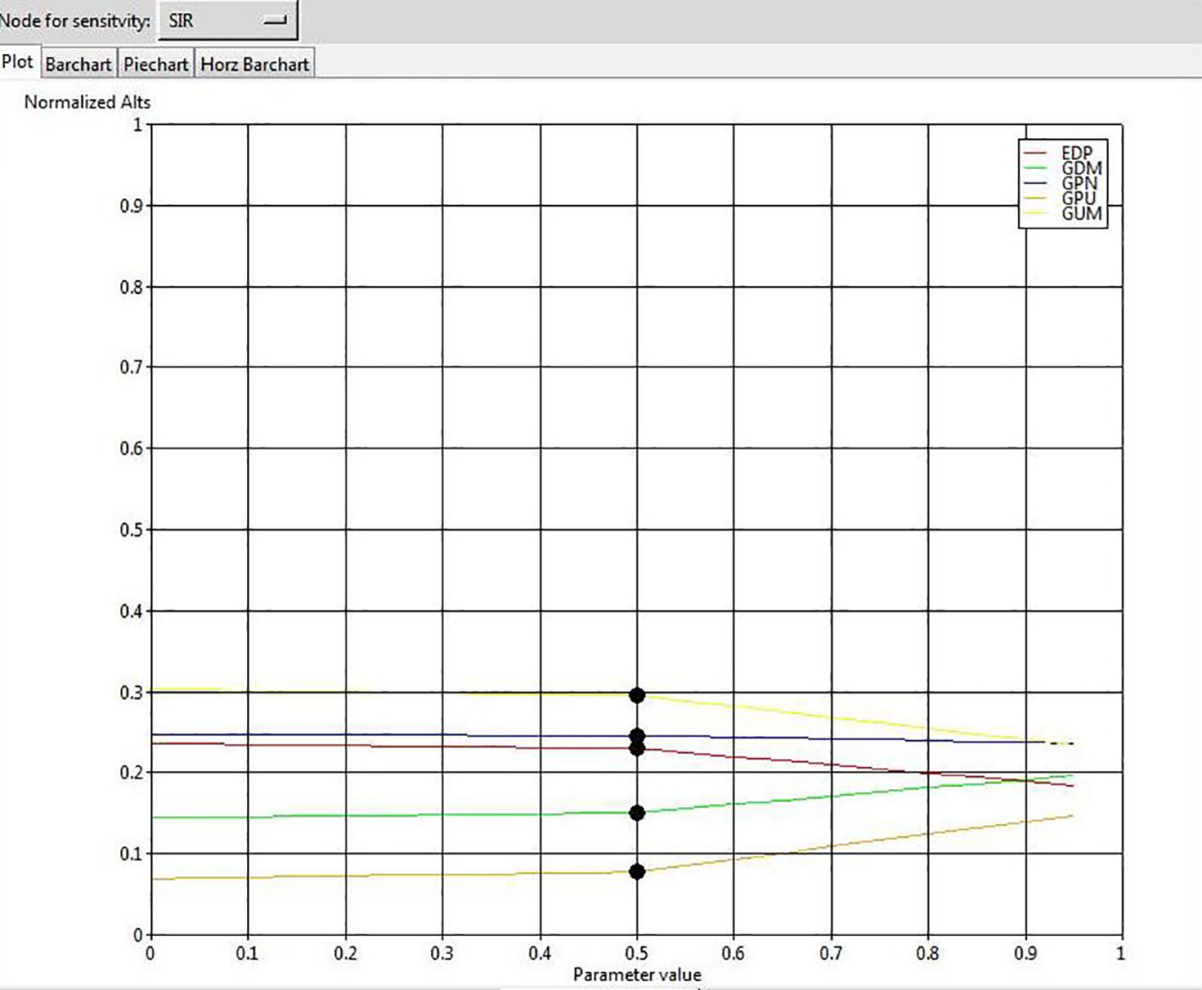
The results of sensitivity analysis for the node “Stakeholder Integration”.

**Fig 7:**
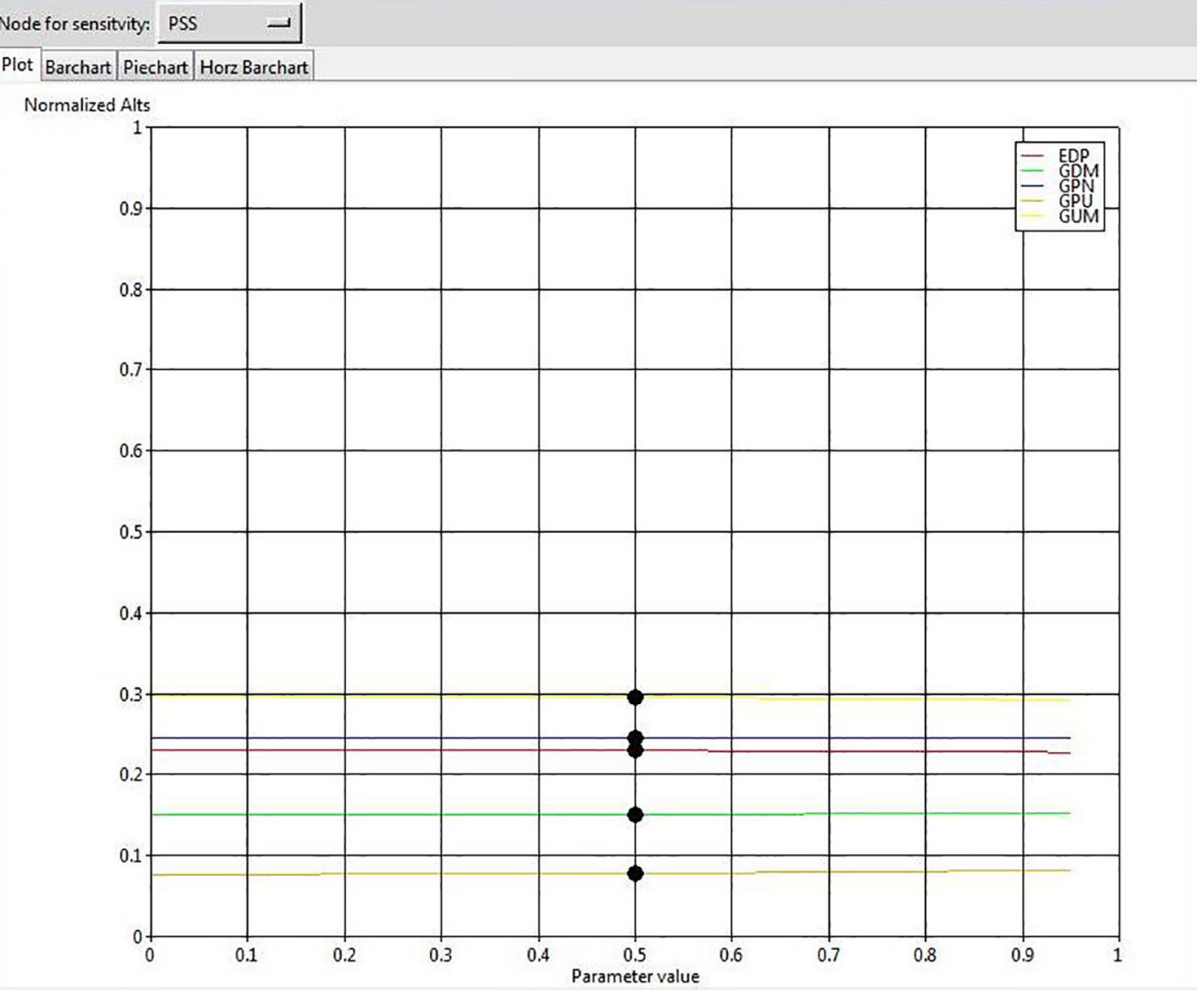
The results of sensitivity analysis for the node “Product Stewardship”.

To ensure the stability of our final outcome, we conducted a sensitivity analysis. The results show that changes in the input parameters by the value of 70% do not affect the overall rank of GSCIs, confirming that the solution is robust. For example, the results of sensitivity analysis for 4 elements are illustrated in Figs 4, 5, 6, and 7.

#### Computing the performance

To complete our analysis and finalise the priorities, we calculated the performance of each GSCI in company X. For this purpose, we asked the company to make a score between 0 (not at all), and 100 (fully implemented) for each indicator associated with the green initiatives. Then, we calculated the performance of the GSCIs by applying the Eq (1). The indicators and their relative weights are given in Table 11. The weights were extracted from the measurement model of the final causal relationship model, calculated by using the software SmartPLS.

$${\text{Performance GSCI}}_{\text{i}}=\sum_{j=1}^{m}\;Score\;I_{ij}*\;Weight\;I_{ij}$$ (1)

where i = 1,…5; and m = Total numbers of the indicators in variable GSCI_i._

**Table 11:** Performance measurement indicators and their relative weights.

Operational Area			Performance Measures	Weights
**Green**	**Eco-Product Design**		1. Design of products for reduced consumption of materials.	0.078281
**Supply**			2. Design of products for reduced consumption of energy.	0.229461
**Chain**			3. Design of products for reuse, recycling, or recovery of materials/components.	0.174082
**Operational**			4. Design of products to avoid or reduce use of hazardous material in products or in their manufacturing processes.	0.341189
**(GSCO)**			5. Product design considering product life cycle costs.	0.176987
**Performance**	**Greening**	Green Purchasing	1. Use of environmental-friendly raw materials (recyclable/renewable) in products.	0.224774
	**upstream**	(0.573547)	2. Substitution of polluting and hazardous materials/parts.	0.238483
			3. Reducing scarce resource usage in products.	0.536742
		Green Supplier Management	1. Supplier selection considering environmental criteria.	0.37031
		(0.426453)	2. Providing support for suppliers to establish and implement their own green programs.	0.248753
			3. Collaboration with suppliers for planning and implementing green.	0.271822
			4. Drive the suppliers to increase their environmental responsiveness.	0.109115
	**Greening Production**		1. Optimisation of manufacturing processes to reduce solid wastes.	0.199082
			2. Optimisation of manufacturing processes for reduced consumption of energy.	0.095561
			3. Optimisation of manufacturing processes to reduce water wastes.	0.006246
			4. Optimisation of manufacturing processes to reduce air emissions.	0.176794
			5. Process design focused on using renewable/recyclable materials.	0.212784
			6. Process design focused on using renewable energy.	0.174789
			7. Recycling of materials internally in the company.	0.134744
	**Greening**	Green Distribution	1. Eco labelling of products.	0.080777
	**Downstream**	(0.399107)	2. Environmental improvement in packaging.	0.496973
			3. Change for more environmentally friendly transportation.	0.42225
		Green Customer Management	1. Providing information to consumers on environment-friendly products.	0.329149
		(0.600893)	2. Cooperation with customer for product eco-design.	0.04903
			3. Cooperation with customers for cleaner production.	0.106448
			4. Cooperation with customers for green packaging.	0.065802
			5. Cooperation with customers for using less energy during product transportation.	0.158998
			6. Cooperation with customers for environmental friendly use of products.	0.290573
	**Greening**	Packaging Recovery	1. Collecting used packaging from customers for reuse or recycling.	0.263962
	**Post-Use**	(0.019003)	2. Returning the packaging of suppliers' products to them for reuse or recycling.	0.438686
			3. Recycling of packages.	0.297352
		Product Recovery	1. Collecting used products from customers for recycling, reclamation, or reuse.	0.148374
		(0.382307)	2. Returning products to suppliers for recycling, retaining of materials, or remanufacturing.	0.178752
			3. Recovering from used or defective products/components (i.e., remanufacturing, repair, rework, or refurbishing).	0.294075
			4. Recycling from End-of-Life products/components.	0.3788
		Investment Recovery	1. Use of recycled materials or used/recovered components in new products.	0.434876
		(0.598689)	2. Use of rebuilt or remanufactured parts for the purpose of after-sales services.	0.310782
			3. Sale of scrap or used materials.	0.088806
			4. Sale of recycled materials or recovered parts.	0.165537

#### Importance-Performance analysis

**Fig 8:**
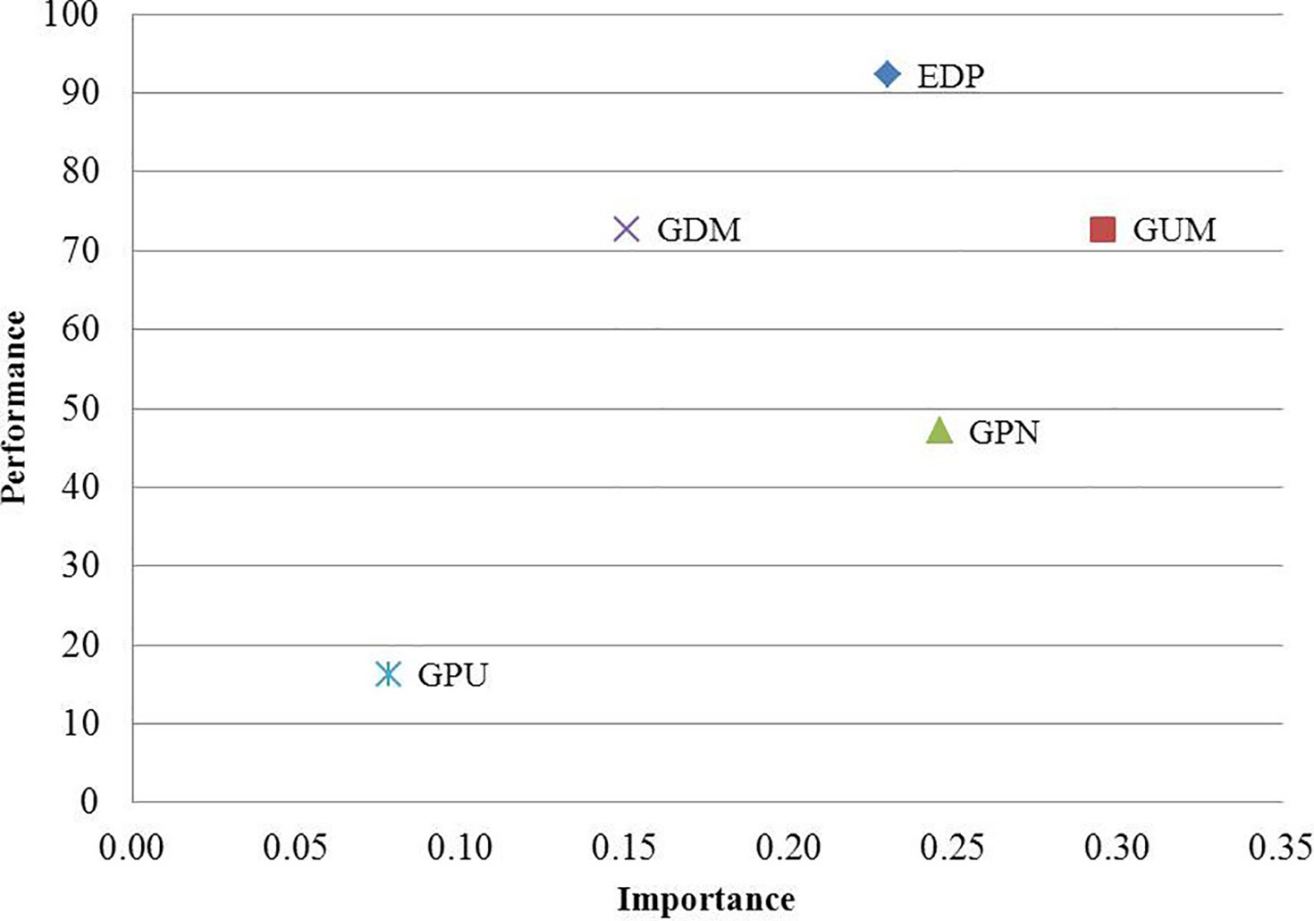
Importance-performance analysis of green supply chain initiatives.

By combining the results of the importance and performance analyses, we can obtain the optimal solution for company X. For this purpose, we multiplied the value of “opportunity for improvement” for each green initiative (that is, one hundred minus the value of the performance) with its value of importance and calculated the contribution of the green initiatives in achieving the prioritised green strategies. Fig 8 illustrates the importance-performance matrix. The results of the final ranking of the green initiatives are given in Table 12. The highest rank goes to the green production initiative, with a value of 39.19% contribution in improving the company’s performance in green strategy adoption. Product design for the environment accounts for the lowest rank, with a value of 5.28%.

**Table 12:** The final ranking of green initiatives in company X.

Green supply chain initiatives	I^1^	P^2^	P*I^3^	O^4^	O*I^5^	TC^6^
**Design for the environment**	0.23	92.28	21.22	7.72	1.78	5.28%
**Greening upstream**	0.29	72.67	21.07	27.33	7.93	23.52%
**Green production**	0.25	47.14	11.79	52.86	13.21	39.19%
**Greening downstream**	0.15	72.71	10.91	27.29	4.09	12.13%
**Greening post-use**	0.08	16.23	1.30	83.77	6.70	19.88%
**Sum**			66.29		33.71	

^1^ Importance

^2^ Performance

^3^ Performance*Importance

^4^ Opportunity for improvement

^5^ Opportunity for improvement* Importance

^6^ Total contribution in improvement opportunities

Each of green initiatives prioritised in this step has several sub-practices (See Table 11). The priorities for these various practices in each GSCI can be obtained by multiplying the value of “opportunity for improvement” for each indicator with its relative weights, presented in Table 11.

The final list of prioritised green strategies, GSCIs, and the various practices in each initiative can help company X initiate its strategic plan for its environmental improvement program. For example because the two highest ranks are assigned to the area of green production and greening upstream, improving these areas can be a good start for improving the firm’s green supplier management. Given the high priority of greening upstream, the company is recommended to pay close attention to expanding the extent of environmental actions to its supplier. Communicating a shared vision among the firm’s suppliers toward the pollution prevention strategy as the first-ranked green strategy is suggested as the starting point. The prioritised list of practices in the area of greening upstream also can be considered a guideline for developing the detailed environmental assessment program for suppliers.

## Evaluation of the method’s usability

To evaluate the usability of the proposed method for the strategic prioritisation of green supply chain initiatives, the EMR in the case study were asked to comment on the entire method and then to give her feedback overall and for each dimension of usability, namely, learnability, helpfulness and efficiency. Her comments and feedback are transcribed and analysed here under.

From the evaluator’ overall opinion about the entire system, it can be seen that she found the method helpful. In addition, she believed that the proposed method is comprehensive, systematic and structured, and involves comprehensive strategic factors and green supply chain initiatives. Regarding the applicability of the outputs, she believed that the outputs are applicable and are consistent with her expectations.

*“The decision factors considered in this software are very comprehensive” “This method systematically makes the priorities*, *now we make decisions based on one-to-one factor*, *but this software compares between several strategic factors and gives us the priorities*.*”*

*“The results go along with what I understand the way the company gives priorities too*. *So*, *the understanding I have now about what the company directs me to do*, *is supported*, *it goes along the same line as the report*, *it doesn’t go against*. *If it is just produces different report I would be shocked*, *but from my experience it seems consistent*. *I mean from my understanding about the companies’ road versus the output of the method*, *the results are consistent with my expectations*.*”*

Regarding the method’s learnability, the expert user concurred that the method is easy to learn and understandable.

*“Regarding the learnability there is no problem*, *actually it is not very complicated”*

From the summary of the feedback obtained, it is found that the evaluator believes that the proposed method helps her to do her job more effectively. She found the method useful for gap analysis and prioritising their environmental improvement opportunities. She also commented that using this method strengthens the decision making process and increases her confidence level while making decisions. She also believed that the data-driven nature of this method provides her with the ability to make solid justification while suggesting environmental improvement programmes to her top managers.

*“This method is useful for us if we want to see where we are now*, *what we want is also there*, *that is good*. *From this method we can identify our weaknesses*, *from the prioritised list of initiatives we know what we should do*, *so we can improve ourselves in that area*. *It helps us to come up with some ideas for improvement of our environmental performance*.*”*

*“It is just a check and balance to see that you are right on track*, *you may know that your main things are logically this*, *this*, *…*, *but this method actually proves*, *it can prove it using numerical data and calculation*, *so it helps to further strengthen your decisions*.*”*

*“When we use this method if somebody asks why these priorities*, *we know that these priorities are made based on comprehensive strategic factors and we can justify that these priorities are based on this*, *this*, *…*. *Now our justification for giving the priority is not very systematic*. *But*, *with this method*, *because the solution is based on statistics*, *we have quantitative evidence and it is much easier to explain to people rather than qualitative justification*. *Qualitative justification always leads to arguments*. *Justification is very important for us*, *because top management ask for justification because the implementation of these initiatives is costly*.*”*

The evaluator also gave good commendations concerning the system efficiency. She believed that the system is efficient in terms of the output she received as compared with the time she spent to implement the method and the volume of input she provided to get the results.

*“It is quiet efficient in a couple of hours we can get a comprehensive report*.*”*

*“I do find that it is very useful because you can do a lot of things with this*, *it doesn’t demand a lot of inputs and it produces a lot of data for you and you can get a lot of useful information from it*.*”*

## Conclusions and Implications for Future Research

By linking the findings from the empirical study of Malaysian industries to the formal assessments of green strategies and GSCIs offered in an ANP decision framework, we developed a practical tool to help company managers develop a strategic goal-oriented plan for their environmentally related programs.

First, this tool determines the priorities of green strategies by identifying managers’ preferences with respect to satisfying external pressures and the desired competitive advantages while considering the firm’s capability to implement these strategies. Second, the GSCIs will be prioritised according to their relative importance to the adoption of the prioritised strategies. Third, regarding the firm’s current performance in each green initiative, the final priorities for improving these performances will be calculated.

There are several opportunities to improve this work as stated below:

- The list of decision factors offered in this study is derived from the literature review. This list can be extended by conducting qualitative research among practitioners who are experts in this research area.
- The environmental management representatives (EMR) who were requested to complete the questionnaire in this research were introduced in the literature as a key informant person in the area of green supply chain management. It is assumed that EMRs have insights into the company’s strategies and policies. However, it is suggested that ANP questionnaire and the part of the survey questionnaire that relates to the firm’s strategy are completed by a involving the firm’s top manager. This might improve the accuracy of the answers. Due to the limitation of the sample size for every specific industry, we used the commutative data collected from multiple industries in our empirical study. Conducting an empirical study for every particular industry can provide an opportunity to developing a more accurate and reliable tool for every specific industry.
- The indicators for evaluating the performance of GSCIs have been proposed based on the various studies on the green supply chain. These indicators can be customised for each particular industry by studying the environmental reports of the leading companies in each industry.
- With respect to the ANP technique and performance measurement system, the nature of the present study leaves room for the application of fuzzy methods in the form of linguistic variables for pairwise comparison and performance metric evaluation.

## Supporting Information

S1 Appendix The questionnaire for cluster comparisons with respect to the company’s business strategy. (DOCX)

S2 Appendix The questionnaire for comparisons with respect to the company’s business strategy in competitive values cluster. (DOCX)

S3 Appendix The questionnaire for comparisons with respect to the company’s business strategy in internal resources cluster. (DOCX)

S4 Appendix The questionnaire for comparisons with respect to company’s business strategy in external pressures cluster. (DOCX)

S5 Appendix Pairwise comparison for clusters IP, CA, and KR and calculation of their relative weights. (DOCX)

S6 Appendix Pairwise comparison for the elements in cluster IP and calculation of their relative weights. (DOCX)

S7 Appendix Pairwise comparison for the elements in cluster CA and calculation of their relative weights. (DOCX)

S8 Appendix Pairwise comparison for the elements in cluster KR and calculation of their relative weights. (DOCX)
