# Supplementary material for: The Development of a Strategic Prioritisation Method for Green Supply Chain Initiatives
Source: PLoS One. 2015 Nov 30;10(11):e0143115. doi: 10.1371/journal.pone.0143115 (PMC4664245; doi:10.1371/journal.pone.0143115)
Supplement: S1 Appendix — (DOCX) [file pone.0143115.s001.docx]

S1 Appendix. Cluster comparisons with respect to the company’s business strategy

| According to your company’s business strategy and policies, please make pairwise comparison of the clusters while planning the environmental strategic plan | | | | | | | | | | | | | | | | | | |
| --- | --- | --- | --- | --- | --- | --- | --- | --- | --- | --- | --- | --- | --- | --- | --- | --- | --- | --- |
| Cluster | Intensity | | | | | | | | | | | | | | | | | Cluster |
|  | 9 | 8 | 7 | 6 | 5 | 4 | 3 | 2 | 1 | 2 | 3 | 4 | 5 | 6 | 7 | 8 | 9 |  |
| Responding to the firm’s external pressures (IP) |  |  |  |  |  |  |  |  |  |  |  |  |  |  |  |  |  | Achieving the firm’s competitive advantages (CA) |
| Responding to the firm’s external pressures (IP) |  |  |  |  |  |  |  |  |  |  |  |  |  |  |  |  |  | Optimum use of available resources (KR) |
| Achieving the firm’s competitive advantages (CA) |  |  |  |  |  |  |  |  |  |  |  |  |  |  |  |  |  | Optimum use of available resources (KR) |
