# Supplementary material for: The Development of a Strategic Prioritisation Method for Green Supply Chain Initiatives
Source: PLoS One. 2015 Nov 30;10(11):e0143115. doi: 10.1371/journal.pone.0143115 (PMC4664245; doi:10.1371/journal.pone.0143115)
Supplement: S2 Appendix — (DOCX) [file pone.0143115.s002.docx]

S2 Appendix. Comparisons with respect to the company’s business strategy in competitive values cluster

| According to your company’s business strategy and policies, please make pairwise comparison of the elements for achieving the firm’s desired competitive advantages while planning the environmental strategic plan | | | | | | | | | | | | | | | | | | |
| --- | --- | --- | --- | --- | --- | --- | --- | --- | --- | --- | --- | --- | --- | --- | --- | --- | --- | --- |
| Element | Intensity | | | | | | | | | | | | | | | | | Element |
|  | 9 | 8 | 7 | 6 | 5 | 4 | 3 | 2 | 1 | 2 | 3 | 4 | 5 | 6 | 7 | 8 | 9 |  |
| Cost reduction (CRA) |  |  |  |  |  |  |  |  |  |  |  |  |  |  |  |  |  | Reputation & legitimacy (RLA) |
| Cost reduction (CRA) |  |  |  |  |  |  |  |  |  |  |  |  |  |  |  |  |  | Future positioning (FPA) |
| Reputation & legitimacy (RLA) |  |  |  |  |  |  |  |  |  |  |  |  |  |  |  |  |  | Future positioning (FPA) |
