# Supplementary material for: The Development of a Strategic Prioritisation Method for Green Supply Chain Initiatives
Source: PLoS One. 2015 Nov 30;10(11):e0143115. doi: 10.1371/journal.pone.0143115 (PMC4664245; doi:10.1371/journal.pone.0143115)
Supplement: S3 Appendix — (DOCX) [file pone.0143115.s003.docx]

S3 Appendix. Comparisons with respect to the company’s business strategy in internal resources cluster

| According to your company’s business strategy and policies, please make pairwise comparison of the elements for optimum use of the firm’s resources while planning environmental strategic plan | | | | | | | | | | | | | | | | | | |
| --- | --- | --- | --- | --- | --- | --- | --- | --- | --- | --- | --- | --- | --- | --- | --- | --- | --- | --- |
| Element | Intensity | | | | | | | | | | | | | | | | | Element |
|  | 9 | 8 | 7 | 6 | 5 | 4 | 3 | 2 | 1 | 2 | 3 | 4 | 5 | 6 | 7 | 8 | 9 |  |
| Continuous improvement (CIR) |  |  |  |  |  |  |  |  |  |  |  |  |  |  |  |  |  | Stakeholder integration (SIR) |
| Continuous improvement (CIR) |  |  |  |  |  |  |  |  |  |  |  |  |  |  |  |  |  | Disruptive change (DCR) |
| Stakeholder integration (SIR) |  |  |  |  |  |  |  |  |  |  |  |  |  |  |  |  |  | Disruptive change (DCR) |
