# Supplementary material for: The Development of a Strategic Prioritisation Method for Green Supply Chain Initiatives
Source: PLoS One. 2015 Nov 30;10(11):e0143115. doi: 10.1371/journal.pone.0143115 (PMC4664245; doi:10.1371/journal.pone.0143115)
Supplement: S4 Appendix — (DOCX) [file pone.0143115.s004.docx]

S4 Appendix. Comparisons with respect to company’s business strategy in external pressures cluster

| According to your company’s business strategy and policies, please make pairwise comparison of the elements for responding to the firm’s external pressures while planning the environmental strategic plan | | | | | | | | | | | | | | | | | | |
| --- | --- | --- | --- | --- | --- | --- | --- | --- | --- | --- | --- | --- | --- | --- | --- | --- | --- | --- |
| Element | Intensity | | | | | | | | | | | | | | | | | Element |
|  | 9 | 8 | 7 | 6 | 5 | 4 | 3 | 2 | 1 | 2 | 3 | 4 | 5 | 6 | 7 | 8 | 9 |  |
| Regulatory pressures (RIP) |  |  |  |  |  |  |  |  |  |  |  |  |  |  |  |  |  | Customer pressures (MIP) |
| Regulatory pressures (RIP) |  |  |  |  |  |  |  |  |  |  |  |  |  |  |  |  |  | Competitor pressures (CIP) |
| Customer pressures (MIP) |  |  |  |  |  |  |  |  |  |  |  |  |  |  |  |  |  | Competitor pressures (CIP) |
