# Supplementary material for: The Development of a Strategic Prioritisation Method for Green Supply Chain Initiatives
Source: PLoS One. 2015 Nov 30;10(11):e0143115. doi: 10.1371/journal.pone.0143115 (PMC4664245; doi:10.1371/journal.pone.0143115)
Supplement: S5 Appendix — (DOCX) [file pone.0143115.s005.docx]

**S5 Appendix. Pairwise comparison for clusters IP, CA, and KR and calculation of their relative weights**

| Goal | IP | CA | KR | **→** |  | Goal |
| --- | --- | --- | --- | --- | --- | --- |
| IP | 1 | 1/4 | 1/3 |  | IP | W1= 0.121957 |
| CA | 4 | 1 | 2 |  | CA | W2= 0.558425 |
| KR | 3 | 1/2 | 1 |  | KR | W3= 0.319618 |

C.R.: 0.01759
