# Supplementary material for: The Development of a Strategic Prioritisation Method for Green Supply Chain Initiatives
Source: PLoS One. 2015 Nov 30;10(11):e0143115. doi: 10.1371/journal.pone.0143115 (PMC4664245; doi:10.1371/journal.pone.0143115)
Supplement: S6 Appendix — (DOCX) [file pone.0143115.s006.docx]

**S6 Appendix. Pairwise comparison for the elements in cluster IP and calculation of their relative weights**

| Goal | RIP | MIP | CIP | **→** |  | Goal |
| --- | --- | --- | --- | --- | --- | --- |
| RIP | 1 | 1 | 1 |  | RIP | W11= 0.32879 |
| MIP | 1 | 1 | 1/2 |  | MIP | W12= 0.26505 |
| CIP | 1 | 2 | 1 |  | CIP | W13= 0.406616 |

C.R.: 0.00000
