# Supplementary material for: The Development of a Strategic Prioritisation Method for Green Supply Chain Initiatives
Source: PLoS One. 2015 Nov 30;10(11):e0143115. doi: 10.1371/journal.pone.0143115 (PMC4664245; doi:10.1371/journal.pone.0143115)
Supplement: S7 Appendix — (DOCX) [file pone.0143115.s007.docx]

**S7 Appendix. Pairwise comparison for the elements in cluster CA and calculation of their relative weights**

| Goal | CRA | RLA | FPA | **→** |  | Goal |
| --- | --- | --- | --- | --- | --- | --- |
| CRA | 1 | 1 | 1 |  | CRA | W21= 0.33333 |
| RLA | 1 | 1 | 1 |  | RLA | W22= 0.33333 |
| FPA | 1 | 1 | 1 |  | FPA | W23= 0.33333 |

C.R.: 0.00000
