# Supplementary material for: The Development of a Strategic Prioritisation Method for Green Supply Chain Initiatives
Source: PLoS One. 2015 Nov 30;10(11):e0143115. doi: 10.1371/journal.pone.0143115 (PMC4664245; doi:10.1371/journal.pone.0143115)
Supplement: S8 Appendix — (DOCX) [file pone.0143115.s008.docx]

**S8 Appendix. Pairwise comparison for the elements in cluster KR and calculation of their relative weights**

| Goal | CIR | SIR | DCR | **→** |  | Goal |
| --- | --- | --- | --- | --- | --- | --- |
| CIR | 1 | 1 | 1 |  | CIR | W31= 0.33333 |
| SIR | 1 | 1 | 1 |  | SIR | W31= 0.33333 |
| DCR | 1 | 1 | 1 |  | DCR | W31= 0.33333 |

C.R.: 0.00000
